# Multiply robust estimation of marginal structural models in observational studies subject to covariate-driven observations

**DOI:** 10.1093/biomtc/ujae065

**Published:** 2024-07-16

**Authors:** Janie Coulombe, Shu Yang

**Affiliations:** Department of Mathematics and Statistics, Université de Montréal, Montreal, Quebec H3T 1J4, Canada; Department of Statistics, North Carolina State University, Raleigh, NC 27607, United States

**Keywords:** average treatment effect, confounding, efficiency, irregular visits, robustness to model misspecification

## Abstract

Electronic health records and other sources of observational data are increasingly used for drawing causal inferences. The estimation of a causal effect using these data not meant for research purposes is subject to confounding and irregularly-spaced covariate-driven observation times affecting the inference. A doubly-weighted estimator accounting for these features has previously been proposed that relies on the correct specification of two nuisance models used for the weights. In this work, we propose a novel consistent multiply robust estimator and demonstrate analytically and in comprehensive simulation studies that it is more flexible and more efficient than the only alternative estimator proposed for the same setting. It is further applied to data from the *Add Health* study in the United States to estimate the causal effect of therapy counseling on alcohol consumption in American adolescents.

## 1 INTRODUCTION

The study of causes and effects is an essential component of learning healthcare systems (Krumholz, 2014). Estimated causal effects, instead of associations, should be used to inform treatment decisions. This manuscript proposes a novel, multiply robust efficient estimator for the marginal causal effect of a treatment that may vary in time on a longitudinal outcome using observational data. Randomized controlled trials are the gold standard for causal inference. Randomization of the treatment options makes patients more comparable in terms of their baseline characteristics. Randomized studies also often have clear protocols for the timing of patients’ visits (ie, observation times) at which patient health status is measured. It is not always possible to conduct a randomized controlled trial designed to answer a specific causal question and researchers often turn to observational data (Black, 1996). We herein focus on the particular features of electronic health records (EHRs) data.

While EHRs are increasingly available for analysis, they are not collected for research purposes. The treatments measured in EHRs are not randomized to patients. This can lead to spurious associations in the data called *confounding* (Greenland and Morgenstern, 2001). These data are also measured irregularly across patients. Each patient follows their pattern in how they access care, also likely to depend on their characteristics. For example, as in Bůžková and Lumley (2005), suppose an observational study in which we are interested in the causal effect of air pollution on forced expiratory volume (FEV). We assume the effect of air pollution on FEV is further mediated by asthma, and that both air pollution and asthma affect the chance for FEV to be measured. Or, as in our application in this manuscript, suppose we aim to estimate the marginal causal effect of therapy on the average number of alcoholic beverages consumed in American adolescents followed irregularly over time, where observation depends on adolescents’ characteristics. Statistically, this creates a long-term dependence structure between the outcome and the visit processes that can result in biased estimators of causal or associational parameters (see eg, Lin and Ying 2001; McCulloch et al. 2016 and most recently Coulombe et al. 2021, 2022; Yang 2022 and Pullenayegum et al. 2023 in a context of causal inference). When aiming for a causal effect, this bias can be due to confounding by the visit process or, if the visit indicators act as colliders (ie, are affected by the treatment prescribed and the study outcome), to collider-stratification bias (Greenland, 2003). In the examples listed above, the causal relationship between air pollution and FEV measurements or therapy and alcohol consumption is also likely confounded.

Under a set of causal and modeling assumptions, causal effects can be inferred by estimating the parameters of a marginal structural model (MSM) fitted on the data from a pseudo-population that is free of confounding and other types of spurious associations, such as collider-stratification bias (Robins et al., 2000). Previous work has tackled this in settings with covariate-driven observation times and confounding, leading to the flexible inverse probability of treatment and monitoring weighted (FIPTM) estimator (Coulombe et al., 2021). For observation times occurring not at random, Pullenayegum et al. (2023) recently proposed another estimator that uses random effects to model the remaining dependence between the observation and the outcome processes. Both methods above may suffer similar issues: They were not developed to be the most efficient estimators in their semiparametric class, and they are not doubly robust, but rather rely on model assumptions for the treatment and the observation times. Yang et al. (2020) and Rytgaard et al. (2023) also proposed semiparametric approaches for the time-specific intervention effects. Their approaches were proposed for the study of survival outcomes as opposed to continuous outcomes. Yang (2022) and Rytgaard et al. (2022) proposed general semiparametric frameworks for estimating intervention-specific mean outcomes. Most of these approaches allow several longitudinal processes in the estimation (exposure, outcome, and covariates) to be measured sporadically by jointly modeling all their observation processes. They are highly flexible but the estimands and estimation approaches used by these authors are different than ours. They focus on the mean outcome difference over a pre-specified period of time, under a specific treatment regime. We herein propose a straightforward, estimating equation approach for the average treatment effect estimated with repeated measurements for which R code, available with the manuscript, is straightforward to implement. We focus on the situation when the observation times occur “at random” (as opposed to completely at random or not at random). The FIPTM estimator can be used in that setting, but it relies on the correct specification of the treatment and outcome observation models as a function of patient characteristics, and can be severely biased when one or both models are not correctly specified. Secondly, the FIPTM could be made more efficient by deriving the influence curve for the causal effect of interest (Tsiatis, 2006). To address these issues, we propose the first multiply robust estimator for the causal marginal effect of a binary treatment on a longitudinal continuous outcome, that accounts for confounding and irregular covariate-driven observation times of the outcome simultaneously. The notation, estimand, causal assumptions, and proposed estimator are presented in Section 2. Simulation studies covering several different scenarios of data generating mechanism (DGM) are presented in Section 3. Our methodology is applied to data from the *Add Health* study in Section 4 and we conclude in Section 5.

## 2 METHODS

### 2.1 Notation

**FIGURE 1:**
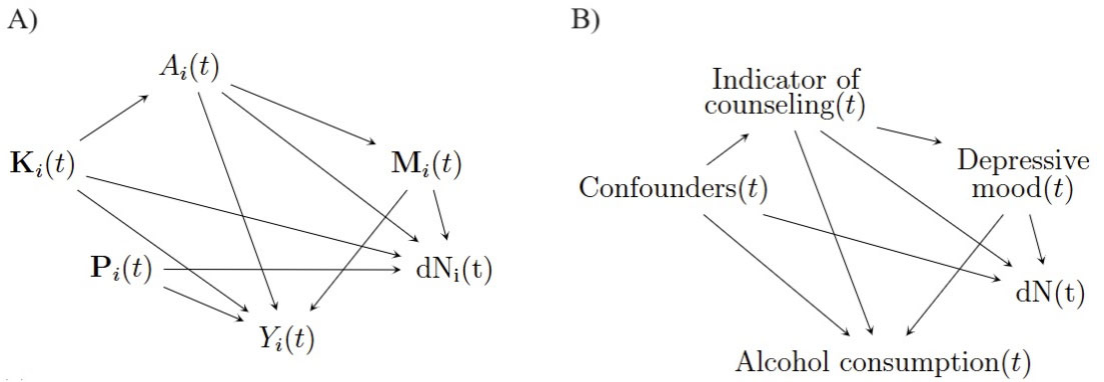
Causal diagram illustrating the assumed DGM. (A) Assumed causal diagram at time $t$ in patient $i$, postulated to be common across all patients. (B) Causal diagram for the Add Health study data at time $t$ common across all adolescents.

We assume working with a random sample of size $n$ from a larger population, $i$ denotes the patient index and $t \in [0, \tau ]$ is the time with $\tau$ a maximum censoring time in the cohort. Let $A_i(t)$ represent the binary treatment taking values in $\left\lbrace 0,1\right\rbrace$ and $Y_i(t)$ be the continuous outcome for patient $i$ at time $t$. We denote vectors and matrices in bold. The type of DGM we focus on is presented in the left panel of Figure 1, in which $\mathbf {K}_i(t)$ are potential confounders for the treatment-outcome relationship (Pearl, 2009), $\mathbf {M}_i(t)$ are potential mediators for the treatment effect on the outcome, and $\mathbf {P}_i(t)$ contains pure predictors of the outcome that could also affect the observation of a patient outcome $Y_i(t)$. The set $\mathbf {P}_i(t)$ is distinguished from $\mathbf {K}_i(t)$ as it could contain visit predictors generated and measured after the treatment. Only the outcome process is assumed to be measured sporadically (eg, the weight is measured irregularly according to patient characteristics such as a change in medication). All the other variables necessary to the estimation of the marginal causal effect of treatment are assumed to be available at all times during follow-up; in EHRs, the drugs and comorbidities are often recorded anytime there is a new diagnosis or a new prescription, so it is often a reasonable assumption. Let $N_i(t)$ be a counting process for observation times of the outcome $Y_i(t)$ between times 0 and $t$ for individual $i$. The indicator ${\rm {d}}N_i(t)$ equals to 1 when there is an observation of the outcome $Y_i(t)$, and 0 otherwise. The set $\mathbf {V}_i(t)$ includes all the variables causing observation times (ie, causing $\rm {d}N_i(t)$) and *also* includes all the confounders of the treatment-outcome relationship. We must include $\mathbf {K}_i(t)$ in the set $\mathbf {V}_i(t)$ for the proposed estimator to be consistent. In Figure 1, we have $\mathbf {V}_i(t)=\lbrace A_i(t), \mathbf {K}_i(t), \mathbf {M}_i(t), \mathbf {P}_i(t) \rbrace$.

Patients are allowed to have different censoring times $C_i$. Denote $\xi _i(t)=\mathbf {1}\lbrace C_i> t \rbrace$ an indicator of patient $i$ still in the study at time $t$. We assume that censoring times are non-informative, an assumption denoted by $Y_i(t) \perp C_i \mid A_i(t)$ (this is discussed more in Section 2.5).

### 2.2 Causal estimand

The potential outcome framework (Neyman, 1923; Rubin, 1976) is used to define our estimand. Denote by $Y^1_i(t)$ and $Y^0_i(t)$ the potential outcomes of individual $i$ at time $t$ if they received treatment option $A_i(t)=1$ or $A_i(t)=0$, respectively. The treatment may be fixed at baseline or it could vary in time. In what follows, we define $A_i(t)$ to be the treatment given at time $t$. The causal marginal effect of the binary treatment on a continuous outcome is defined as $\beta _1=\mathbb {E}\left[ Y^1_i(t) - Y^0_i(t) \right]$. Our interest lies in a cross-sectional effect $\beta _1$ that does not vary in time, which can be estimated using an MSM with which we assume a constant effect (see the Discussion section in which this assumption is discussed).

Suppose a certain time discretization for which there can be only one jump in the counting process $N(t)$ (for instance, daily visits at the doctor’s office). If we had access to all potential outcomes under both treatments and at each time $t$ for the time granularity chosen above, for a random sample of participants of size $n$, we could estimate $\beta _1$ using sample means. On the other hand, by conducting a randomized controlled trial and randomly allocating patients to one of the two treatment options, and observing patients at prespecified visit times, then patients allocated to treatment 1 and treatment 0 should not differ before receiving the treatment. Then, a MSM $\mathbb {E}[Y_i^a(t)\mid A_i(t)=a]= \xi _i(t; \beta _a) = \beta _0 +\beta _1 a$, with $\beta _a= [\beta _0 \beta _1]^T$ could be used to estimate $\beta _1$ without accounting for confounding. In observational data from EHRs, unfortunately, we tend to observe the potential outcomes $Y^1_i(t)$ in those who had greater chances of being treated with $A_i(t)=1$, and the potential outcomes $Y^0_i(t)$ in those who had greater chances of being treated with $A_i(t)=0$ (as a consequence, $\mathbb {E}\left[ Y^a_i(t) \mid A_i(t)=a \right] \ne \mathbb {E}\left[ Y^a_i(t)\right]$). In addition, the potential outcomes for an individual $i$ are only observed at times $t$ when $\rm {d}N_i(t)=1$, which may depend on covariates. We do not have access to all potential outcomes and require causal assumptions to equate the estimand to functions of $Y_i(t)$.

### 2.3 Causal assumptions

Five causal assumptions are required for consistent estimation of the causal marginal effect of treatment (Table 1 corresponding to assumptions 1–3b below). Modeling assumptions for the MSM and the nuisance models are also required, the latter are discussed in Section 2.5.

Outcome consistency, i.e., $Y_i(t)=A_i(t) Y^1_i(t) + \lbrace 1-A_i(t) \rbrace Y^0_i(t)$.
(a) positivity of treatment, meaning that anyone should have a chance of receiving any of the two treatment options, and (b) positivity of observation, such that patients had a chance to have their outcome observed at any time given their characteristics.
Conditional exchangeability, with includes (a) no unmeasured confounder in the observed set $\mathbf {K}_i(t)$; and (b) independence of the observation indicators with other variables in the analysis conditional on the visit predictors $\mathbf {V}_i(t)$.

**TABLE 1:** Causal assumptions required for the proposed estimator to be consistent.

Assumption	Definition
Outcome consistency	$Y_i(t) = A_i(t) Y_i^1(t) + \left\lbrace 1-A_i(t)\right\rbrace Y_i^0(t)$
Positivity of treatment	$0 < \mathbb {P}\lbrace A_i(t)\mid \mathbf {K}_i(t) \rbrace < 1$
Positivity of observation	$0 < \mathbb {E}[\rm {d}N_i(t)\mid \mathbf {V}_i(t)]$
No unmeasured confounder	$\left\lbrace Y_i^0(t), Y_i^1(t)\right\rbrace \perp A_i(t) \mid \mathbf {K}_i(t)$
Conditional exchangeability	$\left\lbrace Y_i^0(t), Y_i^1(t)\right\rbrace \perp A_i(t) \mid \mathbf {K}_i(t)$ and $\lbrace A_i(t), Y^0_i(t), Y^1_i(t)\rbrace \bot \rm {d}N_i(t) \mid \mathbf {V}_i(t)$

These five assumptions must hold, both for the former FIPTM estimator and for the novel proposed estimator to be consistent, except for the inclusion of $\mathbf {K}_i(t)$ in the set $\mathbf {V}_i(t)$ that is only required for the proposed estimator. The conditional exchangeability can be recovered by breaking the spurious associations due to the treatment and observation mechanisms via inverse weights (marginal approach), conditioning on the sets $A_i(t)$ and $\mathbf {V}_i(t)$ (which includes $\mathbf {K}_i(t)$) in a regression model for the outcome and using methods such as g-computation (Robins, 1986) (standardization approach), or as we propose, using both approaches simultaneously to obtain a robust estimator. We review the previously proposed FIPTM estimator.

### 2.4 Previous estimator

Using the marginal approach corresponds to using the FIPTM estimator proposed in Coulombe et al. (2021). It consists of a doubly-weighted least squares estimator that incorporates inverse probability of treatment (IPT) weights (Horvitz and Thompson, 1952; Rosenbaum and Rubin, 1983) and inverse intensity of visit (IIV) weights (Lin et al., 2004). The IPT weights are functions of the confounders $\mathbf {K}_i(t)$ and the IIV weights are functions of the visit predictors $\mathbf {V}_i(t)$. The estimator is consistent for $\beta _1$ when both weights are correctly specified. A parametric model can be used for $\mathbb {P}\lbrace A_{i}(t)=a\mid \mathbf {K}_{i}(t);\boldsymbol {\psi }\rbrace$ to obtain the IPT weights


(1) \begin{eqnarray*}
\mathbf {1}\lbrace A_i(t)=a\rbrace /\mathbb {P}\lbrace A_{i}(t)=a\mid \mathbf {K}_{i}(t);\boldsymbol {\psi }\rbrace,
\end{eqnarray*}

where $\mathbb {P}\lbrace A_{i}(t)=1\mid \mathbf {K}_{i}(t);\boldsymbol {\psi }\rbrace$, the propensity score, is the probability of receiving the treatment 1 as a function of predictors $\mathbf {K}_{i}(t)$ and parameters $\boldsymbol {\psi }$ (Rosenbaum and Rubin, 1983). A logistic regression can be used to compute an estimated propensity score. The IIV weights can be obtained by modeling the mean visit indicator as a function of covariates $\mathbf {V}_i(t).$ Since the visit indicator is binary and visits are recurrent, one can use a model for recurrent visits such as the Andersen and Gill (1982) model (which corresponds to a proportional rate model) or a logistic regression model. Both models rely on relatively similar assumptions for the mean visit indicator when using the same set of covariates, but the proportional rate model models the rate and the logistic regression, the probability of visit. They lead to similar estimates of the rate and probability of visit when the visits are rare such that only one visit occurs over a time unit (eg, a day), see e.g., Papoulis and Pillai (2002). The proportional rate model for the visits as a function of $\mathbf {V}_i(t)$ is given by


(2) \begin{eqnarray*}
\mathbb {E}[ {\rm {d}}N_{i}(t)\mid \mathbf {V}_{i}(t); \boldsymbol {\gamma }]=\xi _{i}(t)\exp \lbrace \boldsymbol {\gamma }^{\mathrm{\scriptscriptstyle T}}\mathbf {V}_{i}(t)\rbrace \lambda _{0}(t){\rm {d}}t.
\end{eqnarray*}

The baseline rate of observation $\lambda _{0}(t)$ in (2) consists of the visit rate when all variables $\mathbf {V}_i(t)$ are set to their reference level. With the FIPTM estimator, the baseline rate can be removed from the IIV weights without affecting the marginal effect of treatment estimate since this would still make the weights in (3) proportional to the intensity of being observed as a function of $\mathbf {V}_{i}(t)$. The IIV weights can also be stabilized, in which case the baseline rate cancels automatically in the weights and need not be estimated (Bůžková and Lumley, 2009). This leads to the following intensity of visit weights (which we will take the inverse of), from which $\boldsymbol {\gamma }$ parameters can be estimated using the Andersen and Gill (1982) model:


(3) \begin{eqnarray*}
\mathbb {E}[{\rm {d}}N_i(t) \mid \mathbf {V}_i(t); \boldsymbol {\gamma } ]= \exp \lbrace \boldsymbol {\gamma }^{\mathrm{\scriptscriptstyle T}}\mathbf {V}_{i}(t)\rbrace .
\end{eqnarray*}

In simulation studies, we assessed both the logistic regression and the proportional rate model. Then, the FIPTM estimator solves the following equations:


(4) \begin{eqnarray*}
&&\mathbb {E}_{n}\left[\int _{0}^{\tau }\frac{\frac{\mathbf {1}\lbrace A_{i}(t)=a\rbrace }{\mathbb {P}\lbrace A_{i}(t)=a\mid \mathbf {K}_{i}(t);\widehat{\boldsymbol {\psi }}\rbrace }Y_{i}(t)-\zeta _{i}(t;\beta _{a})}{\mathbb {E}\lbrace {\rm {d}}N_i(t) \mid \mathbf {V}_i(t); \widehat{\boldsymbol {\gamma }} \rbrace }{\rm {d}}N_{i}(t)\right]\\
&&\qquad=0, a\in \lbrace 0, 1\rbrace ,
\end{eqnarray*}

where $\mathbb {E}_n$ stands for the empirical mean. However, that estimator requires both the treatment and the observation models to be correctly specified, which is not easy in practice.

### 2.5 Novel estimator

We propose the augmented AAIIW estimator (which acronym stands for *doubly augmented and doubly inverse weighted*) that is more flexible and allows two out of four different models to be misspecified while the estimator remains consistent. The estimator is developed by finding the influence curve of the estimand introduced in Section 2.4 (Hines et al., 2022). The novel estimator is obtained by solving the following augmented versions of (4):


(5) \begin{eqnarray*}
& & \mathbb {E}_n \left[\int _{0}^{\tau }\frac{ \eta _i(t) }{\mathbb {E}\lbrace {\rm {d}}N_i(t) \mid \mathbf {V}_i(t); \widehat{\boldsymbol {\gamma }} \rbrace }{\rm {d}}N_{i}(t)\right] \\
&&\quad- \mathbb {E}_n \left[ \int _{0}^{\tau }\frac{{\rm {d}}M_{i}(t) \mathbb {E}\lbrace \eta _i(t)\mid A_i(t)=a, \mathbf {K}_i(t), \mathbf {V}_i(t) \rbrace }{\mathbb {E}\lbrace {\rm {d}}N_i(t) \mid \mathbf {V}_i(t); \widehat{\boldsymbol {\gamma }} \rbrace } \right] \\
& &\quad =0,
\end{eqnarray*}

where the nuisance terms can, for instance, be estimated using parametric models, with


\begin{eqnarray*}
\eta _i(t) &= &\frac{\mathbf {1}\lbrace A_{i}(t)=a\rbrace }{\mathbb {P}\lbrace A_{i}(t)=a\mid \mathbf {K}_{i}(t);\widehat{\boldsymbol {\psi }}\rbrace }Y_{i}(t)\\
&&\quad{-\frac{\mathbf {1}\lbrace A_{i}(t)=a\rbrace -\mathbb {P}\lbrace A_{i}(t)=a\mid \mathbf {K}_{i}(t);\widehat{\boldsymbol {\psi }}\rbrace }{\mathbb {P}\lbrace A_{i}(t)=a\mid \mathbf {K}_{i}(t);\widehat{\boldsymbol {\psi }}\rbrace } \mu _{a}\lbrace \mathbf {K}_{i}(t);\widehat{\boldsymbol {\alpha }}_{K}\rbrace }\\
&&-\zeta _{i}(t;\beta _{a}),
\end{eqnarray*}

with ${\rm {d}}M_{i}(t)={\rm {d}}N_{i}(t)-\xi _{i}(t)\exp \lbrace \widehat{\boldsymbol {\gamma }}^{\mathrm{\scriptscriptstyle T}}\mathbf {V}_{i}(t)\rbrace \widehat{\lambda }_{0}(t){\rm {d}}t$ the martingale residual for the observation process. The conditional outcome mean models in the augmented terms are $\mu _a\left\lbrace \mathbf {K}_i(t); \boldsymbol {\alpha }_K\right\rbrace =\mathbb {E}[Y_i(t)\mid A_i(t)=a, \mathbf {K}_i(t); \boldsymbol {\alpha }_K]$ and $\mu _a\left\lbrace \mathbf {V}_i(t); \boldsymbol {\alpha }_V\right\rbrace =\mathbb {E}[Y_i(t)\mid A_i(t)=a, \mathbf {V}_i(t) ; \boldsymbol {\alpha }_V]$. The latter model arises when taking the expectation $\mathbb {E}\left[ Y_i(t) \mid A_i(t)=a, \mathbf {V}_i(t)\right]$ in the term $\mathbb {E}\lbrace \eta _i(t)\mid A_i(t)=a, \mathbf {K}_i(t), \mathbf {V}_i(t) \rbrace$ in equation (5). For the novel estimator, if using the proportional rate model for visits, then the baseline rate $\lambda _{0}(t)$ in (2) must be estimated before calculating the IIV weights, which was not the case with the FIPTM estimator. The IIV weights in the equations for the AAIIW are the inverse of $\mathbb {E}[\rm {d}N_i(t) \mid \mathbf {V}_i(t); \widehat{\boldsymbol {\gamma }} ]=\widehat{\lambda }_0(t)\exp \lbrace \widehat{\boldsymbol {\gamma }}^{\mathrm{\scriptscriptstyle T}}\mathbf {V}_{i}(t)\rbrace$. One can use the Breslow’s estimator (Cox, 1972) (which we use in our simulation studies)


\begin{eqnarray*}
\widehat{\lambda }_0(t) = \frac{ \sum _{i=1}^n \mathbf {1}\lbrace {\rm {d}}N_i(t)=1 \rbrace }{ \sum _{i=1}^n \mathbf {1}\lbrace {\rm {d}}N_i(t)=1\rbrace \exp \left\lbrace \widehat{\boldsymbol {\gamma }}^{T} \mathbf {V}_i(t) \right\rbrace } .
\end{eqnarray*}

Table 2 shows the combinations of correctly specified models leading to a consistent AAIIW estimator. At least one of the two models related to confounders and one of the two models related to the observation predictors must be correctly specified. The estimand has an efficient influence function if it is pathwise differentiable, i.e., if the univariate submodels are smooth in the parameters, for the postulated models (see eg, Hines et al., 2022). We herein assume that the causal marginal effect of treatment is pathwise differentiable. The derivation of the estimator using the theory of influence functions and a proof of multiple robustness are in Web Appendices A and B, respectively. The link between the theory of influence functions and the theory on model-assisted estimation for our proposed estimator is in Web Appendix C. The correct specification for parametric models is elaborated in Web Appendix D.

**TABLE 2:** Multiple robustness of AAIIW: AAIIW is consistent under Scenarios (a)–(d): $\checkmark$ means correctly specified and $X$ means no requirement.

Scenario	$\mathbb {P}\lbrace A_{i}(t)=a\mid \mathbf {K}_{i}(t);\boldsymbol {\psi } \rbrace$	$\mu _{a}\lbrace \mathbf {K}_{i}(t);\boldsymbol {\alpha }_{K}\rbrace$	$\mathbb {E}\left[\rm {d}N_i(t)=1\mid \mathbf {V}_i(t);\boldsymbol {\gamma }\right]$	$\mu _{a}\lbrace \mathbf {V}_{i}(t);\boldsymbol {\alpha }_{V}\rbrace$
(a)	$\checkmark$	$X$	$\checkmark$	$X$
(b)	$X$	$\checkmark$	$X$	$\checkmark$
(c)	$X$	$\checkmark$	$\checkmark$	$X$
(d)	$\checkmark$	$X$	$X$	$\checkmark$

We show in Web Appendix E that the AAIIW asymptotic variance, derived as the variance of its influence function (Tsiatis, 2006), is smaller than that of the FIPTM when all nuisance models are correctly specified for both estimators. In practice, the AAIIW can be obtained by estimating the nuisance models parameters using e.g., logistic regressions for the treatment and visit models or a proportional rate model for the visits with coxph in R and two linear models for the outcome conditional on $A_i(t)$ and $\mathbf {K}_i(t)$ or $\mathbf {V}_i(t)$. The estimates can be plugged into the estimating equations of the AAIIW. Root solvers (such as uniroot in R) can be used to estimate $\beta _0$ and that estimate be plugged into the second equation, solved for $\beta _1$.

In Web Appendix F, we propose to relax the assumption that censoring occurs at random and to use inverse probability of censoring weights (IPCW) (Robins and Finkelstein, 2000) to address informative censoring. We further outline a multiply robust approach considering censoring predictors. The scenario with informative censoring is also assessed in simulations.

## 3 SIMULATION STUDY

In simulation studies, we compared four different estimators detailed in Table 3.

**TABLE 3:** Estimators compared in simulation studies.

Estimator	$\mathbb {P}\lbrace A_{i}(t)=a\mid \mathbf {K}_{i} (t);\boldsymbol {\psi } \rbrace$	$\mu _{a}\lbrace \mathbf {K}_{i}(t);\boldsymbol {\alpha }_{K}\rbrace$	$\mathbb {E}\left[{\rm {d}}N_i(t)=1\mid \mathbf {V}_i(t);\boldsymbol {\gamma }\right]$	$\mu _{a}\lbrace \mathbf {V}_{i}(t);\boldsymbol {\alpha }_{V}\rbrace$
OLS				
IPT$_c$	$\checkmark$			
IPT$_{nc}$	$\dagger$			
DW$_{c}$	$\checkmark$		$\checkmark$	
DW$_{iptc}$	$\checkmark$		$\dagger$	
DW$_{iivc}$	$\dagger$		$\checkmark$	
DW$_{nc}$	$\dagger$		$\dagger$	
AAIIW$_c$	$\checkmark$	$\checkmark$	$\checkmark$	$\checkmark$
AAIIW$_{s.a}$	$\checkmark$	$\dagger$	$\checkmark$	$\dagger$
AAIIW$_{s.b}$	$\dagger$	$\checkmark$	$\dagger$	$\checkmark$
AAIIW$_{s.c}$	$\dagger$	$\checkmark$	$\checkmark$	$\dagger$
AAIIW$_{s.d}$	$\checkmark$	$\dagger$	$\dagger$	$\checkmark$

OLS is an ordinary least squares estimator, IPT is an IPT-weighted estimator, DW is the (doubly-weighted) FIPTM estimator from Coulombe et al. (2021) and AAIIW is the novel proposed estimator. A $\checkmark$ means correctly specified and a $\dagger$ symbol means it is used as a nuisance model in the estimator but it is wrongly specified.

The DGM was strongly inspired by similar simulation studies presented in Bůžková and Lumley (2009), Coulombe et al. (2021, 2022) and is detailed more thoroughly in Web Appendix G. The DGM included a set of confounders at baseline repeated through follow-up, a time-varying binary treatment, a set of observation predictors that varied in time, and irregular observation of the outcome. The causal effect of treatment was constant, i.e., we correctly specified the MSM in our simulations. The main results for 1000 simulations using a nonhomogeneous Poisson rate to simulate the observation times of the outcome and a sample of size 1000 are presented in the following Section 3.1. In another set of simulations, we replaced the nonhomogeneous Poisson rate with a nonhomogeneous Bernoulli probability for the observation indicator and used a logistic regression instead of the Andersen and Gill model to fit the probability of observation at each time point. These results are presented in Web Appendix H (Web Figures 2 and 3), along with the results under a sample of size 250 instead of 1000 (Web Figure 1) and all Monte Carlo biases and mean square errors (Web Table 1). In both settings using either the Poisson rate of the Bernoulli probability, we tested four different sets of $\boldsymbol {\gamma }$ parameters in the observation model, including one set of zeros (which we call “set 1” in the results), corresponding to uninformative observation. In another sensitivity analysis, we assessed the performance of the proposed estimator under informative censoring that depends on the visit predictors $\mathbf {V}_i(t)$. We compared IPC-weighted and more naive estimators that do not address censoring. The simulation setup is described in Web Appendix G and the results from that analysis (empirical bias and mean squared error) are shown in Web Table 2 (Web Appendix H) and briefly discussed in Section 3.1.

### 3.1 Results

**FIGURE 2:**
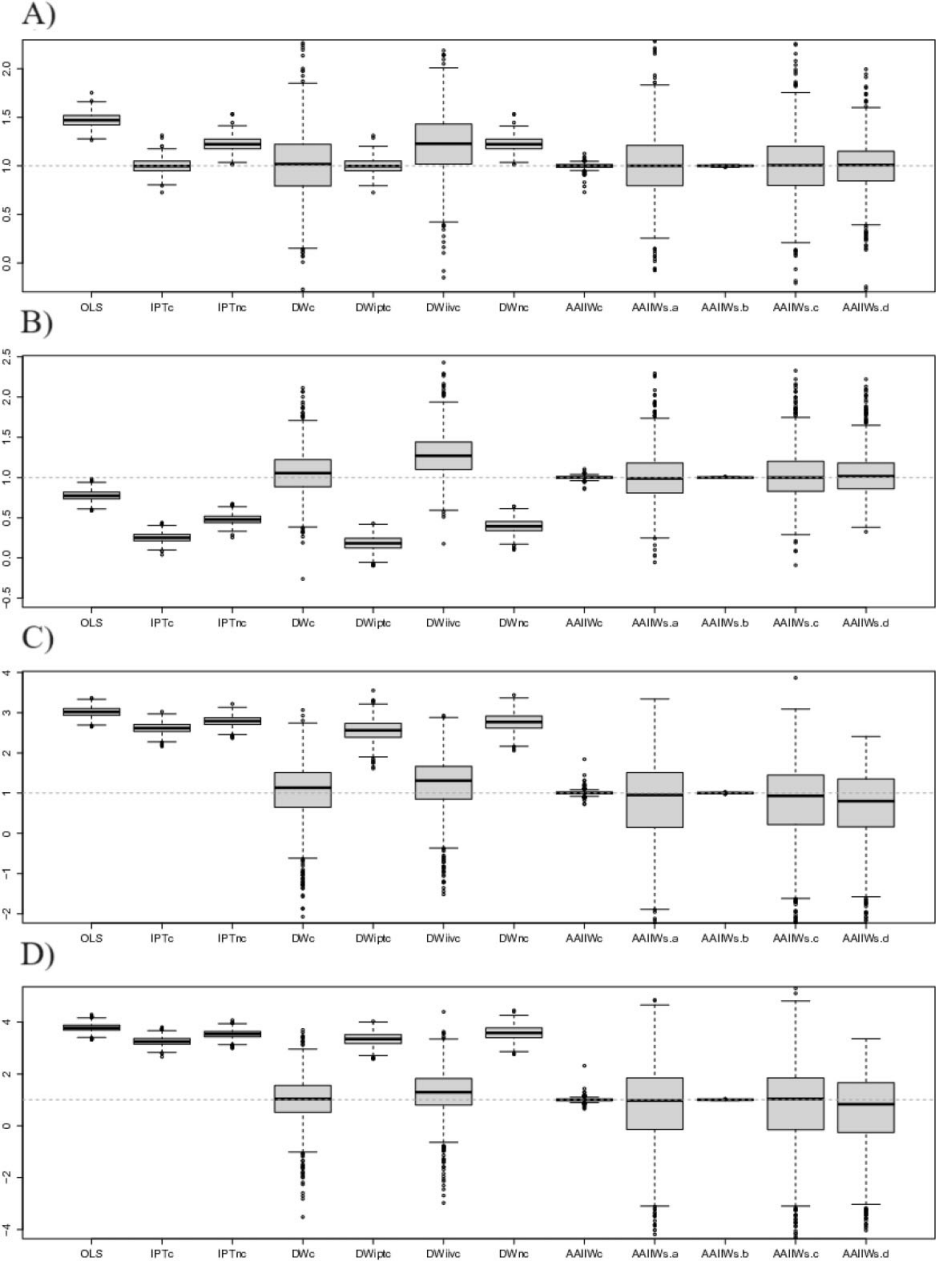
Results of the simulation studies with a sample size of 1000 using a nonhomogeneous Poisson rate to simulate the observation indicators and the Andersen and Gill model with Breslow estimator to estimate the IIV weights. Each boxplot represents the distribution of 1000 estimates for the corresponding estimator. The dashed line represents the gold standard, i.e., the true value for the marginal effect of exposure that equals to 1. Different strengths of the visit process on covariates are represented with scenarios (A) $\gamma =(0, 0, 0, 0, 0, -5)$ (ie, no bias due to the visit process expected); (B) $\gamma =(0.5, 0.3, -0.5, -2, 0, -3)$; (C) $\gamma =(0.5, -0.5, -0.2, -1, 1, -3)$; and (D) $\gamma =(-1, -0.8, 0.1, 0.3, -1, -3)$. OLS, ordinary least squares; IPT, inverse probability of treatment weights; and DW, doubly-weighted estimator which corresponds to the FIPTM from Coulombe et al. (2021); AAIIW: The novel doubly augmented, doubly weighted estimator. The subscripts $c$, $nc$, $iptc$, and $iivc$, respectively mean all correct, all not correct, only IPT correct, and only IIV correct in the nuisance models. The subscripts $s.a$ to $s.d$ refer to scenarios (A)–(D) in Table 2 of the manuscript.

The distributions of 1000 estimates obtained with each estimator using a sample of size 1000 patients are presented in Figure 2. A thorough discussion of the results is given in Web Appendix H with more details on the performance of each of the more naive estimators.

The results are generally as expected. The AAIIW estimator is empirically unbiased in all scenarios (1)–(4) for the observation process, whenever using one of the four combinations of correctly specified models shown in Table 2 or when all four models are correctly specified. It exhibits small variance when the two conditional outcome mean models are correctly specified (scenario b from Table 2) or, as expected when all four models are correctly specified. Results for the second set of simulations using the Bernoulli probability to simulate the observations, and those for a sample of size 250 are in Web Appendix H. As expected, the estimators are more variable when using a sample size of 250, although the same patterns in the comparison of estimators are observed (Web Figure 1). Similar results are observed when using the Bernoulli probability instead of the Poisson rate for the simulation of observation indicators (Web Figures 3 and 3). The simulations using the Bernoulli probability did not require the use of Breslow’s estimator for the baseline rate, which may partly explain the smaller variances observed overall (eg, compare Figure 1 and Web Figure 2).

Results for the DGM with informative censoring that depended on the predictors of visit were also as expected (Web Table 2, Web Appendix H). The informative censoring did affect the empirical bias in some of the scenarios tested. Adjustment via IPCW brought the estimates closer to the true causal effect, with a maximum bias that went from 0.31 to 0.14 after adjustment, for the AAIIW estimator. The AAIIW estimator coupled with IPCW performed particularly well when the two outcome conditional mean models were correctly specified, or when the outcome model conditional on the confounders and the IIV weights were correctly specified (bias smaller than 0.02 in all scenarios).

## 4 MOTIVATING EXAMPLE

We applied the proposed AAIIW estimator and different more naive comparators to longitudinal data from the *Add Health* study in the United States (Harris and Udry, 2022). More details on that study and the analysis are available in Web Appendix I. We have access to data from the first four waves of the *Add Health* study, corresponding to the years 1994–1995, 1996, 2001–2002, and 2008–2009, respectively. Various types of information, including demographics and health status variables were collected via questionnaires filled by the American adolescents in this study. Our goal was to estimate the marginal causal effect of counseling on alcohol consumption based on the question *In the past year, have you received psychological or emotional counseling?*. The assumed DGM is shown in Figure 1. Two challenges we wanted to consider in the analysis are the irregular observation of the outcome and, because the study is observational, the potential confounding of the psychotherapy–alcohol consumption relationship. We selected several potential confounders for that relationship (Web Appendix I). The analysis dataset contained several missing values. We used multiple imputations by chained equations (Rubin, 1988) five times, to impute missing values in covariates. The outcome was defined using the question *Think of all the times you had a drink during the past 12 months. How many drinks did you usually have each time?*. It consists of a self-assessed number of drinks the adolescent would consume, on average, each time they consumed alcohol, ranging from 0 to 90. In this application, the outcome was assessed at each of the four waves for everyone (ie, it contained no missing value). To assess the advantage of our approach, we simulated missingness in the outcome and assessed the different estimators in that setting, knowing the true underlying missingness mechanism. Assuming that all potential confounders as well as the mediator (depressive mood) and the exposure (counseling) affect the chance of observing the alcohol consumption outcome, the outcome observation (ie, the opposite of missingness) was simulated using a pre-specified, invented model (Web Appendix I). We conducted the analysis using each of the five imputed datasets one by one. We used Rubin’s rule (Rubin, 1976) to combine the final estimates from all the estimators compared, and 500 bootstrap samples to obtain confidence intervals (CI). We fit a propensity score model and two different proportional rate models for the observation of the outcome, one correctly specified and one that was not correctly specified (as a function of the sinus of age and depressive mood only).

An ordinary least squares estimator, an IIV-weighted estimator that accounts for the observation process (we tested the two sets of the IIV weights), a doubly-weighted estimator corresponding to the FIPTM estimator (incorporating the IPT weights based on our assumptions on the potential confounders, and IIV weights—we tested the two sets of IIV weights), and the AAIIW estimator in which we incorporated the IPT weights and the two different sets of the IIV weights were compared. We also added a complete data analysis in which an OLS, an IPT-weighted and an augmented inverse probability of treatment weighted (AIPW) estimators were computed on the dataset with no missing data for the outcome.

Some differences were found across the two exposure groups in the first imputed dataset, which indicates potential confounding (Web Appendix J, Web Table 3). In the outcome observation model, we also found modest differences in female sex and smoking status between those for whom the alcohol consumption was observed and the others (Web Appendix J, Web Table 4). After IIV weighting, most differences vanished (Web Table 4).

Both the adjustment for confounding and the one for outcome missingness bring the estimates for the marginal effect of exposure to counseling toward the null. The estimator that led to the closest estimates to the complete data analysis (point estimate 0.35 with the AIPW, Table 4) is the AAIIW estimator, which led to point estimates of 0.40 and 0.39 when using the correct or the wrong IIV weights, respectively. The FIPTM estimator led to point estimates of 0.36 and 0.72, respectively (Table 4), with the estimator using the wrong IIV weights leading to the estimate further away from the gold standard point estimate. Our results indicate that in a setting in which we would not know the true observation mechanism, the AAIIW estimator might still lead to an estimate of the causal effect closer to the complete data analysis, while the FIPTM is more at risk of being biased if its inverse weights are wrongly specified. Our proposed approach allows adjusting for previous (observed) treatments or outcomes as potential confounders or visit predictors, but it cannot address settings in which a previous outcome (that is not observed) affects the observation of any future outcome. In this application, we did not include previous outcomes in the adjustment set for that reason, even if previous outcome values were available. The proposed AAIIW estimator can be used to estimate a time-fixed average treatment effect. In this application, it is possible that the causal effect of therapy on alcohol consumption changes in time, with e.g., a greater benefit of therapy at the beginning of follow-up, but we estimated an “averaged” over all times treatment effect, $\beta _1$. The true treatment effect could vary in time. A lengthier discussion on the study results is given in Web Appendix J.

**TABLE 4:** Complete outcome data (top) and irregularly observed outcome data (bottom) estimates (95% bootstrap percentiles CI) of the marginal effect of counseling on the average number of alcoholic beverages consumed, *Add Health* study, United States, 1996–2008.

Complete data estimates			
OLS	IPT$^\phi$	AIPW$^\phi$	
0.60 (0.41, 0.77)	0.31 (0.16, 0.49)	0.35 (0.20, 0.53)	
Irregularly observed outcome estimates			
OLS	IPT$^\phi$	IIV$^\dagger$	IIV$^\ddagger$
0.86 (0.58, 1.10)	0.57 (0.35, 0.81)	0.68 (-0.32, 1.87)	1.10 (0.62, 1.34)
FIPTM$^{\phi ,\dagger }$	FIPTM$^{\phi ,\ddagger }$	AAIIW$^{\phi ,\dagger }$	AAIIW$^{\phi ,\ddagger }$
0.36 (−0.63, 1.55)	0.72 (0.34, 1.03)	0.40 (−1.36, 2.53)	0.39 (−0.13, 1.34)

Acronyms: CI, confidence interval; IPT, inverse probability of treatment; AIPW, augmented inverse probability of treatment weighted; IIV, inverse intensity of visit; FIPTM, the flexible inverse probability of treatment and monitoring; AAIIW, the doubly augmented, doubly inverse weighted. $^\phi$. Note we do not know the true data generating mechanism for the treatment mechanism in the application. $^\dagger$. This estimator uses a correctly specified generating mechanism for outcome missingness. $^\ddagger$. This estimator uses a wrongly specified generating mechanism for outcome missingness.

## 5 DISCUSSION

This work proposed the first multiply robust estimator for the causal marginal effect of treatment addressing confounding and irregular visits, that is consistent when only two out of four nuisance models, one related to confounders and one to visit predictors, are correctly specified. In addition to being more robust than the FIPTM, the AAIIW estimator is also the most efficient estimator in its semiparametric class. In simulation studies, it was demonstrated to be robust and empirically as efficient as the FIPTM when the two weight models are correctly specified but it could be much more efficient in some other scenarios.

In an application to the *Add Health* study in the United States, we found a difference between more naive estimators and the multiply robust AAIIW estimator in the estimation of the causal marginal effect of therapy counseling on alcohol consumption, and the proposed estimator led to the estimates that were the closest to a gold standard found with the complete dataset. It is possible, however, that unmeasured confounding remains. Sensitivity analyses can be used to assess the effect of unmeasured confounding or visit predictors that were not accounted properly in the estimator (see eg, McCulloch and Neuhaus 2020 for diagnostics on visit irregularity when visit times may depend on the outcome values, or VanderWeele and Arah 2011 for sensitivity analyses that address unmeasured confounding).

The consistency of our proposed estimator relies on specific combinations of correctly specified nuisance models listed in Table 2 and some classical causal assumptions mentioned in Section 2, including conditional exchangeability. See Web Appendix K for some recommendations on the identification of adjustment sets. The proposed approach also relies on the assumed MSM. We assume in this work that the outcome is related to the treatment at time $t$ by a constant parameter (causal effect) $\beta _1$. Thus, our working model, the assumed MSM, is only correctly specified if the treatment causal effect is constant, i.e., if it is the same for any time $t$. If it is not, then the estimated effect corresponds to the closest time-fixed effect to the true, time-varying causal effect and acts as a summary of the true causal relationship if all nuisance models are correctly specified (Neugebauer and van der Laan, 2007). Furthermore, if the working MSM model is not correctly specified, causal interpretation is more difficult, as the estimated effect is averaged over all time points and does not represent the causal effect of treatment at time $t$. That effect can instead be interpreted as the average treatment effect over the entire follow-up period, if one followed a constant treatment course ($A_i(t)=1$ for all $t$, or $A_i(t)=0$ for all $t$), but it becomes harder to interpret if one follows a treatment course with treatment switches. In such settings, nonparametric MSM such as proposed in Neugebauer and van der Laan (2007) could be preferable to estimate causal curves as a function of time, or the treatment and the visit processes could be modeled jointly to acknowledge the lack of generalizability of the effect at one time, to other times when there is no visit (see eg, Robins et al. 2008; Neugebauer et al. 2017, who discussed identification of optimal treatment and visit strategies under joint models for the two processes).

## Supplementary Material

ujae065_Supplemental_Files Web Appendices A, B, C, D, E, and F referenced in Section 2, Web Appendices G and H referenced in Section 3, Web Appendices I and J referenced in Section 4, Web Appendix K referenced in Section 5, and the R code to reproduce the simulation studies from Section 3 are available with this paper at the Biometrics website on Oxford Academic.

## Data Availability

The data that support the findings in this paper are from the Add Health program which are available at https://doi.org/10.3886/ICPSR21600.v21. More information on obtaining Add Health data is available on the project website (https://addhealth.cpc.unc.edu).
