# Supplementary material for: Multiply robust estimation of marginal structural models in observational studies subject to covariate-driven observations
Source: Biometrics. 2024 Jul 16;80(3):ujae065. doi: 10.1093/biomtc/ujae065 (PMC11250490; doi:10.1093/biomtc/ujae065)
Supplement: ujae065_Supplemental_Files — Web Appendices A, B, C, D, E, and F referenced in Section 2, Web Appendices G and H referenced in Section 3, Web Appendices I and J referenced in Section 4, Web Appendix K referenced in Section 5, and the R code to reproduce the simulation studies from Section 3 are available with this paper at the Biometrics website on Oxford Academic. [file ujae065_supplemental_files.zip › Appendix final.pdf]

Supplementary Material for “Multiply robust estimation of marginal structural models in observational studies subject to covariate-driven observations”

Janie Coulombe,[1] and Shu Yang.

[1]Corresponding author: Department of Mathematics and Statistics, Université de Montréal, Pavillon André-Aisenstadt (AA-5190), 2920 chemin de la Tour, Montréal (Québec), H3T 1J4. Email: janie.coulombe@umontreal.ca.

Contents

Web Appendix A: Derivation of the AAIIW **2**

Web Appendix B: Proof of consistency of the AAIIW **6**

Web Appendix C: Relation with model-assisted estimation **9**

Web Appendix D: Meaning of a correct specification of a model **10**

Web Appendix E: Relative asymptotic efficiency of AAIIW **11**

Web Appendix F: Addressing informative censoring with the multiply robust estimator . . **16**

Web Appendix G: Additional details on the simulation setup **18**

Web Appendix H: Additional results from simulation studies **20**

Web Appendix I: Additional details on the Add Health Study **26**

Web Appendix J: Additional results in the Add Health Study **28**

Web Appendix K: Recommendations for choosing adjustment sets **30**

Web Appendix A: Derivation of the AAIIW

The functional that interests us is given by

$$\psi = E[Y^1(t) - Y^0(t)].$$

Data are denoted by

$$\mathbf{Z}(t) = \{\mathbf{K}(t), \mathbf{P}(t), A(t), \mathbf{M}(t), dN(t), Y(t)\}.$$

We assume the following non-parametric model (the data generating mechanism is also depicted in the causal diagram in Figure 1 in the main manuscript):

$$P\{\mathbf{Z}(t)\} = P\{\mathbf{K}(t)\}P\{\mathbf{P}(t)\}P\{A(t) \mid \mathbf{K}(t)\}P\{\mathbf{M}(t) \mid A(t)\}P\{Y(t) \mid \mathbf{K}(t), \mathbf{P}(t), A(t), \mathbf{M}(t)\}$$
$$\times P\{dN(t) \mid A(t), \mathbf{M}(t), \mathbf{K}(t), \mathbf{P}(t)\}.$$

In the following demonstration, we further denote:

$$\begin{aligned}
\mathbf{V}(t) =& \{\mathbf{M}(t), \mathbf{P}(t), \mathbf{K}(t)\} \quad \text{the visit predictors without the exposure}\\
\mathbf{V}'(t) =& \{\mathbf{M}(t), \mathbf{P}(t)\} \quad \text{the visit predictors without the exposure and confounders}\\
\mu_a\{\mathbf{v}(t)\} =& E[Y(t) \mid A(t) = a, dN(t) = 1, \mathbf{V}(t) = \mathbf{v}(t)]\\
\mu_a\{\mathbf{k}(t)\} =& E[Y(t) \mid A(t) = a, dN(t) = 1, \mathbf{K}(t) = \mathbf{k}(t)]\\
\pi\{\mathbf{k}(t)\} =& P\{A(t) = 1 \mid \mathbf{K}(t) = \mathbf{k}(t))\}\\
\rho\{\mathbf{v}(t)\} =& P\{dN(t) = 1 \mid \mathbf{V}(t) = \mathbf{v}(t)\}.
\end{aligned}$$

Sometimes we use the notation $P(j)$ to denote $P(J = j)$ more generally. We also use the notation $\mathbb{I}(\cdot)$ for an indicator function.

A first approach discussed by Kennedy (2022) to derive the influence curve is called Strategy 1 and uses Gateaux derivatives to derive the EIF. That approach was used in the work of Kandasamy et al. (2015) and Hines et al. (2022), among others. Kennedy (2022) proposed a second strategy (2) based on derivative rules which basically allows a few shortcuts to Strategy 1 and leads to the efficient influence function in fewer steps. Both strategies can be compared and should lead to the same answer.

We derive the influence curve ($\mathbb{IF}$), which corresponds to the efficient influence function, using Strategy 2, that consists of (1) pretending data are discrete, (2) treating influence functions as derivatives, allowing use of differentiation rules, and (3) using influence function building blocks that are known, i.e., the $\mathbb{IF}$ for $E[Y \mid X = x]$ is given by $\frac{\mathbb{I}(X=x)}{P(X=x)}\{Y - E[Y \mid X = x]\}$ (see Section 3.4.3 in Kennedy, 2022).

We use this approach to derive the influence curve, starting first with the influence curve for the left of ψ, i.e., for $\psi^1 = E[Y^1(t)]$, and obtain the influence curve for $E[Y^0(t)]$ by reproducing similar derivations as below. We have:

$$\begin{aligned}
\psi^1 =& E[Y^1(t)]\\
=& E_{\mathbf{V}(t)}\left[E[Y^1(t) \mid dN(t) = 1, A(t) = 1, \mathbf{V}(t)]\right] \text{ by iterated expectation and A1-A3}\\
=& E_{\mathbf{K}(t),\mathbf{V}'(t)}\left[E[Y^1(t) \mid dN(t) = 1, A(t) = 1, \mathbf{V}'(t), \mathbf{K}(t)]\right] \text{ by separating variables in the set } \mathbf{V}\\
=& E_{\mathbf{K}(t),\mathbf{V}'(t)}\left[\mu_1\{\mathbf{v}(t)\}\right].
\end{aligned}$$

For the influence curve of ψ^1, denoted by $\mathbb{IF}(\psi^1)$, we have

$$\begin{aligned}
\mathbb{IF}(\psi^1) =& \mathbb{IF}\left(\sum_{\mathbf{v}'(t)}\sum_{\mathbf{k}(t)} \mu_1\left\{\mathbf{v}(t)\right\} P\{\mathbf{V}'(t)=\mathbf{v}'(t)\mid A(t)=1,\mathbf{K}(t)=\mathbf{k}(t)\}P\{\mathbf{K}(t)=\mathbf{k}(t)\}\right) \text{ using (1)} \\
=& \sum_{\mathbf{v}'(t)}\sum_{\mathbf{k}(t)} \mathbb{IF}\left(\mu_1\left\{\mathbf{v}(t)\right\}\right) P\{\mathbf{V}'(t)=\mathbf{v}'(t)\mid A(t)=1,\mathbf{K}(t)=\mathbf{k}(t)\}P\{\mathbf{K}(t)=\mathbf{k}(t)\} \\
&+ \sum_{\mathbf{v}'(t)}\sum_{\mathbf{k}(t)} \mu_1\left\{\mathbf{v}(t)\right\} \mathbb{IF}\left(P\{\mathbf{V}'(t)=\mathbf{v}'(t)\mid A(t)=1,\mathbf{K}(t)=\mathbf{k}(t)\}\right) P\{\mathbf{K}(t)=\mathbf{k}(t)\} \\
&+ \sum_{\mathbf{v}'(t)}\sum_{\mathbf{k}(t)} \mu_1\left\{\mathbf{v}(t)\right\} P\{\mathbf{V}'(t)=\mathbf{v}'(t)\mid A(t)=1,\mathbf{K}(t)=\mathbf{k}(t)\}\mathbb{IF}\left(P\{\mathbf{K}(t)=\mathbf{k}(t)\right)\} \text{ using (2).}
\end{aligned}$$

Denote the expressions above by (a), (b), and (c), respectively, for the respective 3 rows. We will expand each term separately, starting first with (a):

$$\begin{aligned}
(a) =& \sum_{\mathbf{v}'(t)}\sum_{\mathbf{k}(t)} \mathbb{IF}\left(\mu_1\left\{\mathbf{v}(t)\right\}\right) P\{\mathbf{V}'(t)=\mathbf{v}'(t)\mid A(t)=1,\mathbf{K}(t)=\mathbf{k}(t)\}P\{\mathbf{K}(t)=\mathbf{k}(t)\} \\
=& \sum_{\mathbf{v}'(t)}\sum_{\mathbf{k}(t)} \frac{\mathbb{I}(A(t)=1,dN(t)=1,\mathbf{V}'(t)=\mathbf{v}'(t),\mathbf{K}'(t)=\mathbf{k}'(t))}{P(A(t)=1,dN(t)=1,\mathbf{V}'(t)=\mathbf{v}'(t),\mathbf{K}'(t)=\mathbf{k}'(t))}\left\{Y(t)-\mu_1\left\{\mathbf{v}(t)\right\}\right\} \\
& \qquad \times P\{\mathbf{V}'(t)=\mathbf{v}'(t)\mid A(t)=1,\mathbf{K}(t)=\mathbf{k}(t)\}P\{\mathbf{K}(t)=\mathbf{k}(t)\} \\
& \text{using strategy (3) from Kennedy (2022)} \\
=& \sum_{\mathbf{v}'(t)}\sum_{\mathbf{k}(t)} \frac{\mathbb{I}(A(t)=1,dN(t)=1,\mathbf{V}'(t)=\mathbf{v}'(t),\mathbf{K}'(t)=\mathbf{k}'(t))\left\{Y(t)-\mu_1\left\{\mathbf{v}(t)\right\}\right\}}{P(A(t)=1\mid\mathbf{k}(t))P(dN(t)=1\mid\mathbf{v}(t))P\{\mathbf{V}'(t)=\mathbf{v}'(t)\mid A(t)=1,\mathbf{K}(t)=\mathbf{k}(t)\}P\{\mathbf{K}(t)=\mathbf{k}(t)\}} \\
& \qquad \times P\{\mathbf{V}'(t)=\mathbf{v}'(t)\mid A(t)=1,\mathbf{K}(t)=\mathbf{k}(t)\}P\{\mathbf{K}(t)=\mathbf{k}(t)\} \\
& \text{where we factorized the joint density of } A(t), dN(t), \mathbf{V}'(t) \text{ and } \mathbf{K}(t)\text{, according to the DGM in Figure 1} \\
=& \sum_{\mathbf{v}'(t)}\sum_{\mathbf{k}(t)} \frac{\mathbb{I}(A(t)=1,dN(t)=1,\mathbf{V}'(t)=\mathbf{v}'(t),\mathbf{K}'(t)=\mathbf{k}'(t))\left\{Y(t)-\mu_1\left\{\mathbf{v}(t)\right\}\right\}}{P(A(t)=1\mid\mathbf{k}(t))P(dN(t)=1\mid\mathbf{v}(t))} \\
=& \sum_{\mathbf{v}'(t)}\sum_{\mathbf{k}(t)} \frac{\mathbb{I}(A(t)=1,dN(t)=1,\mathbf{V}'(t)=\mathbf{v}'(t),\mathbf{K}'(t)=\mathbf{k}'(t))\left\{Y(t)-\mu_1\left\{\mathbf{v}(t)\right\}\right\}}{\pi\{\mathbf{k}(t)\}\rho\left\{\mathbf{v}(t)\right\}} \\
=& \frac{\mathbb{I}(A(t)=1)\mathbb{I}(dN(t)=1)\left\{Y(t)-\mu_1\left\{\mathbf{v}(t)\right\}\right\}}{\pi\{\mathbf{k}(t)\}\rho\left\{\mathbf{v}(t)\right\}}.
\end{aligned}$$

Then, for (b), we have:

$$
\begin{aligned}
(b) =& \sum_{\mathbf{v}'(t)} \sum_{\mathbf{k}(t)} \mu_1 \{\mathbf{v}(t)\} \, \mathbb{IF} \left(P\{\mathbf{V}'(t) = \mathbf{v}'(t) \mid A(t) = 1, \mathbf{K}(t) = \mathbf{k}(t)\} \right) P\{\mathbf{K}(t) = \mathbf{k}(t)\} \\
=& \sum_{\mathbf{v}'(t)} \sum_{\mathbf{k}(t)} \mu_1 \{\mathbf{v}(t)\} \left\{ \frac{\mathbb{I}(A(t) = 1, \mathbf{K}(t) = \mathbf{k}(t))}{P(A(t) = 1, \mathbf{K}(t) = \mathbf{k}(t))} \left[\mathbb{I}(\mathbf{V}'(t) = \mathbf{v}'(t)) - P\{\mathbf{V}'(t) = \mathbf{v}'(t) \mid A(t) = 1, \mathbf{K}(t) = \mathbf{k}(t)\} \right] \right\} \\
& \times P\{\mathbf{K}(t) = \mathbf{k}(t)\} \\
=& \sum_{\mathbf{v}'(t)} \sum_{\mathbf{k}(t)} \mu_1 \{\mathbf{v}(t)\} \left\{ \frac{\mathbb{I}(A(t) = 1, \mathbf{K}(t) = \mathbf{k}(t))}{P(A(t) = 1, \mathbf{K}(t) = \mathbf{k}(t))} \mathbb{I}(\mathbf{V}'(t) = \mathbf{v}'(t)) \right\} P\{\mathbf{K}(t) = \mathbf{k}(t)\} \\
& - \sum_{\mathbf{v}'(t)} \sum_{\mathbf{k}(t)} \mu_1 \{\mathbf{v}(t)\} \left\{ \frac{\mathbb{I}(A(t) = 1, \mathbf{K}(t) = \mathbf{k}(t))}{P(A(t) = 1, \mathbf{K}(t) = \mathbf{k}(t))} P\{\mathbf{V}'(t) = \mathbf{v}'(t) \mid A(t) = 1, \mathbf{K}(t) = \mathbf{k}(t)\} \right\} P\{\mathbf{K}(t) = \mathbf{k}(t)\} \\
=& \sum_{\mathbf{v}'(t)} \sum_{\mathbf{k}(t)} \mu_1 \{\mathbf{v}(t)\} \left\{ \frac{\mathbb{I}(A(t) = 1, \mathbf{K}(t) = \mathbf{k}(t))}{P(A(t) = 1, \mid \mathbf{K}(t) = \mathbf{k}(t)) P(\mathbf{K}(t) = \mathbf{k}(t))} \mathbb{I}(\mathbf{V}'(t) = \mathbf{v}'(t)) \right\} P\{\mathbf{K}(t) = \mathbf{k}(t)\} \\
& - \sum_{\mathbf{v}'(t)} \sum_{\mathbf{k}(t)} \mu_1 \{\mathbf{v}(t)\} \left\{ \frac{\mathbb{I}(A(t) = 1, \mathbf{K}(t) = \mathbf{k}(t))}{P(A(t) = 1 \mid \mathbf{K}(t) = \mathbf{k}(t)) P(\mathbf{K}(t) = \mathbf{k}(t))} P\{\mathbf{V}'(t) = \mathbf{v}'(t) \mid A(t) = 1, \mathbf{K}(t) = \mathbf{k}(t)\} \right\} \\
& P\{\mathbf{K}(t) = \mathbf{k}(t)\} \text{ by factorizing the joint density of treatment and confounders} \\
=& \sum_{\mathbf{v}'(t)} \sum_{\mathbf{k}(t)} \mu_1 \{\mathbf{v}(t)\} \left\{ \frac{\mathbb{I}(A(t) = 1, \mathbf{K}(t) = \mathbf{k}(t), \mathbf{V}'(t) = \mathbf{v}'(t))}{\pi \{\mathbf{k}(t)\}} \right\} \\
& - \sum_{\mathbf{v}'(t)} \sum_{\mathbf{k}(t)} \mu_1 \{\mathbf{v}(t)\} \left\{ \frac{\mathbb{I}(A(t) = 1, \mathbf{K}(t) = \mathbf{k}(t))}{\pi \{\mathbf{k}(t)\}} P\{\mathbf{V}'(t) = \mathbf{v}'(t) \mid A(t) = 1, \mathbf{K}(t) = \mathbf{k}(t)\} \right\} \\
=& \frac{\mu_1 \{\mathbf{v}(t)\}}{\pi \{\mathbf{k}(t)\}} - \frac{\mu_1 \{\mathbf{k}(t)\}}{\pi \{\mathbf{k}(t)\}} \text{ since the } \mathbf{v}'(t) \text{ could be integrated out in the second piece.}
\end{aligned}
$$

and for (c), we have:

$$
\begin{aligned}
(c) =& \sum_{\mathbf{v}'(t)} \sum_{\mathbf{k}(t)} \mu_1 \{\mathbf{v}(t)\} \, P\{\mathbf{V}'(t) = \mathbf{v}'(t) \mid A(t) = 1, \mathbf{K}(t) = \mathbf{k}(t)\} \mathbb{IF} \left(P\{\mathbf{K}(t) = \mathbf{k}(t)) \right\} \\
=& \sum_{\mathbf{v}'(t)} \sum_{\mathbf{k}(t)} \mu_1 \{\mathbf{v}(t)\} \, P\{\mathbf{V}'(t) = \mathbf{v}'(t) \mid A(t) = 1, \mathbf{K}(t) = \mathbf{k}(t)\} \left\{ \mathbb{I}(\mathbf{K}(t) = \mathbf{k}(t)) - P\{\mathbf{K}(t) = \mathbf{k}(t)\} \right\} \\
=& \sum_{\mathbf{v}'(t)} \sum_{\mathbf{k}(t)} \mu_1 \{\mathbf{v}(t)\} \, P\{\mathbf{V}'(t) = \mathbf{v}'(t) \mid A(t) = 1, \mathbf{K}(t) = \mathbf{k}(t)\} \mathbb{I}(\mathbf{K}(t) = \mathbf{k}(t)) \\
& - \sum_{\mathbf{v}'(t)} \sum_{\mathbf{k}(t)} \mu_1 \{\mathbf{v}(t)\} \, P\{\mathbf{V}'(t) = \mathbf{v}'(t) \mid A(t) = 1, \mathbf{K}(t) = \mathbf{k}(t)\} P\{\mathbf{K}(t) = \mathbf{k}(t)\} \\
=& \sum_{\mathbf{v}'(t)} \mu_1 \{\mathbf{v}(t)\} \, P\{\mathbf{V}'(t) = \mathbf{v}'(t) \mid A(t) = 1, \mathbf{K}(t) = \mathbf{k}(t)\} \\
& - \sum_{\mathbf{v}'(t)} \sum_{\mathbf{k}(t)} \mu_1 \{\mathbf{v}(t)\} \, P\{\mathbf{V}'(t) = \mathbf{v}'(t) \mid A(t) = 1, \mathbf{K}(t) = \mathbf{k}(t)\} P\{\mathbf{K}(t) = \mathbf{k}(t)\} \\
=& \mu_1 \{\mathbf{k}(t)\} - \psi^1,
\end{aligned}
$$

since the first term was marginalized over $\mathbf{V}'(t)$ and the second term is merely the estimand.

Putting all the terms together, we obtain the following influence curve for the functional of interest, under the non-parametric model specified above:

$$
\begin{aligned}
\mathbb{IF}(\psi^1) =& (a) + (b) + (c) \\
=& \frac{\mathbb{I}(A(t) = 1) \mathbb{I}(dN(t) = 1) \{Y(t) - \mu_1 \{\mathbf{v}(t)\}\}}{\pi\{\mathbf{k}(t)\} \rho \{\mathbf{v}(t)\}} + \frac{\mu_1 \{\mathbf{v}(t)\}}{\pi \{\mathbf{k}(t)\}} - \frac{\mu_1 \{\mathbf{k}(t)\}}{\pi \{\mathbf{k}(t)\}} + \mu_1 \{\mathbf{k}(t)\} - \psi^1
\end{aligned}
$$

which can be transformed into:

$$\mathbb{IF}(\psi^1) = \frac{\mathbb{I}(A(t)=1)\mathbb{I}(dN(t)=1)Y(t)}{\pi\{\mathbf{k}(t)\}\rho\{\mathbf{v}(t)\}} + \mu_1\{\mathbf{v}(t)\}\left(\frac{1}{\pi\{\mathbf{k}(t)\}} - \frac{\mathbb{I}(A(t)=1)\mathbb{I}(dN(t)=1)}{\pi\{\mathbf{k}(t)\}\rho\{\mathbf{v}(t)\}}\right) + \mu_1\{\mathbf{k}(t)\}\left(1 - \frac{1}{\pi\{\mathbf{k}(t)\}}\right) - \psi^1.$$

corresponding to the estimating equations of the AAIIW estimator where ψ^1 can be replaced by the model postulated for $E[Y^1(t)]$, i.e., $\beta_0 + \beta_1$.

Similarly, for $E[Y^0(t)]$, we obtain

$$\mathbb{IF}(\psi^0) = \frac{\mathbb{I}(A(t)=0)\mathbb{I}(dN(t)=1)Y(t)}{(1-\pi\{\mathbf{k}(t)\})\rho\{\mathbf{v}(t)\}} + \mu_0\{\mathbf{v}(t)\}\left(\frac{1}{(1-\pi\{\mathbf{k}(t)\})} - \frac{\mathbb{I}(A(t)=0)\mathbb{I}(dN(t)=1)}{(1-\pi\{\mathbf{k}(t)\})\rho\{\mathbf{v}(t)\}}\right) + \mu_0\{\mathbf{k}(t)\}\left(1 - \frac{1}{(1-\pi\{\mathbf{k}(t)\})}\right) - \psi^0$$

where ψ^0 can be replaced by the model postulated for $E[Y^0(t)]$, i.e., β_0.

Our proposed AAIIW estimator corresponds to the influence curve, i.e., it has the efficient influence function and is the most locally efficient in its class.

References

Hines, O., Dukes, O., Diaz-Ordaz, K., and Vansteelandt, S. (2022). Demystifying statistical learning based on efficient influence functions. *The American Statistician*, 76(3), pp. 292-304.

Kandasamy, K., Krishnamurthy, A., Poczos, B., Wasserman, L., and Robins, J. M. (2015). Nonparametric von mises estimators for entropies, divergences and mutual informations. *Advances in Neural Information Processing Systems*, 28.

Kennedy, E. H. (2022). Semiparametric doubly robust targeted double machine learning: A review. *arXiv* preprint arXiv:2203.06469.

Web Appendix B: Proof of consistency of the AAIIW

Proof of the multiple robustness of the novel estimator under the different scenarios presented in Table 2 in the main manuscript

The probability limiting estimating equations of the AAIIW estimator are

$$pr\left[\int_0^\tau \frac{\frac{\mathbf{1}\{A_i(t)=a\}}{pr\{A_i(t)=a|\mathbf{K}_i(t);\hat\psi\}}Y_i(t)-\frac{\mathbf{1}\{A_i(t)=a\}-pr\{A_i(t)=a|\mathbf{K}_i(t);\hat\psi\}}{pr\{A_i(t)=a|\mathbf{K}_i(t);\hat\psi\}}\mu_a\{\mathbf{K}_i(t);\hat\alpha_K\}-\zeta_i(t;\beta_a)}{E\{\mathrm{dN_i(t)}=1\mid \mathbf{V}_\mathrm{i}(\mathrm{t});\hat\gamma\}}\mathrm{dN_i(t)}\right]$$
$$-pr\left[\int_0^\tau \frac{\mathrm{dM_i(t)}\left(\frac{\mu_\mathrm{a}\{\mathbf{V}_\mathrm{i}(\mathrm{t});\hat\alpha_\mathrm{V}\}}{\mathrm{pr}\{\mathrm{A_i(t)=a}|\mathbf{K}_\mathrm{i}(\mathrm{t});\hat\psi\}}-\frac{\mathbf{1}\{\mathrm{A_i(t)=a}\}-\mathrm{pr}\{\mathrm{A_i(t)=a}|\mathbf{K}_\mathrm{i}(\mathrm{t});\hat\psi\}}{\mathrm{pr}\{\mathrm{A_i(t)=a}|\mathbf{K}_\mathrm{i}(\mathrm{t});\hat\psi\}}\mu_\mathrm{a}\{\mathbf{K}_\mathrm{i}(\mathrm{t});\hat\alpha_\mathrm{K}\}-\zeta_\mathrm{i}(\mathrm{t};\beta_\mathrm{a})\right)}{E\{\mathrm{dN_i(t)}=1\mid \mathbf{V}_\mathrm{i}(\mathrm{t});\hat\gamma\}}\right]=0.$$

In this proof, denote the correct models using an asterisk onto the parameters, i.e., the correct model for the treatment is denoted by $pr\{A_i(t)=a\mid \mathbf{K}_i(t);\psi^*\}$.

Under scenario a) (Table 2) we have

$$pr\left[\int_0^\tau \frac{\frac{\mathbf{1}\{A_i(t)=a\}}{pr\{A_i(t)=a|\mathbf{K}_i(t);\psi^*\}}Y_i(t)-\frac{\mathbf{1}\{A_i(t)=a\}-pr\{A_i(t)=a|\mathbf{K}_i(t);\psi^*\}}{pr\{A_i(t)=a|\mathbf{K}_i(t);\psi^*\}}\mu_a\{\mathbf{K}_i(t);\hat\alpha_K\}-\zeta_i(t;\beta_a)}{pr\{\mathrm{dN_i(t)}=1\mid \mathbf{V}_\mathrm{i}(\mathrm{t});\gamma^*\}}\mathrm{dN_i(t)}\right]$$
$$-pr\left[\int_0^\tau \frac{(N_i(t)-pr\{\mathrm{dN_i(t)}=1\mid \mathbf{V}_\mathrm{i}(\mathrm{t});\gamma^*\})\left(\frac{\mu_a\{\mathbf{V}_i(t);\hat\alpha_V\}}{pr\{A_i(t)=a|\mathbf{K}_i(t);\psi^*\}}-\frac{\mathbf{1}\{A_i(t)=a\}-pr\{A_i(t)=a|\mathbf{K}_i(t);\psi^*\}}{pr\{A_i(t)=a|\mathbf{K}_i(t);\psi^*\}}\mu_a\{\mathbf{K}_i(t);\hat\alpha_K\}\right)}{pr\{\mathrm{dN_i(t)}=1\mid \mathbf{V}_\mathrm{i}(\mathrm{t});\gamma^*\}}\right]$$
$$+pr\left[\int_0^\tau \frac{(N_i(t)-pr\{\mathrm{dN_i(t)}=1\mid \mathbf{V}_\mathrm{i}(\mathrm{t});\gamma^*\})\zeta_i(t;\beta_a)}{pr\{\mathrm{dN_i(t)}=1\mid \mathbf{V}_\mathrm{i}(\mathrm{t});\gamma^*\}}\right.$$
$$=pr\left[\int_0^\tau \frac{\frac{\mathbf{1}\{A_i(t)=a\}}{pr\{A_i(t)=a|\mathbf{K}_i(t);\psi^*\}}Y_i(t)-\zeta_i(t;\beta_a)}{pr\{\mathrm{dN_i(t)}=1\mid \mathbf{V}_\mathrm{i}(\mathrm{t});\gamma^*\}}\mathrm{dN_i(t)}\right]$$
$$=0$$

which is clearly unbiased for the parameter of interest since both weights are correctly specified in scenario a).

In scenario b) (Table 2), we rewrite the original estimating equations as follows:

$$pr\left[\int_0^\tau \frac{\frac{\mathbf{1}\{A_i(t)=a\}}{pr\{A_i(t)=a|\mathbf{K}_i(t);\hat\psi\}}(Y_i(t)-\mu_a\{\mathbf{K}_i(t);\hat\alpha_K\})+\mu_a\{\mathbf{K}_i(t);\hat\alpha_K\}-\zeta_i(t;\beta_a)}{E\{\mathrm{dN_i(t)}=1\mid \mathbf{V}_\mathrm{i}(\mathrm{t});\hat\gamma\}}\mathrm{dN_i(t)}\right]$$
$$-pr\left[\int_0^\tau \frac{\frac{\mathbf{1}\{A_i(t)=a\}}{pr\{A_i(t)=a|\mathbf{K}_i(t);\hat\psi\}}(\mu_a\{\mathbf{V}_i(t);\hat\alpha_V\}-\mu_a\{\mathbf{K}_i(t);\hat\alpha_K\})+\mu_a\{\mathbf{K}_i(t);\hat\alpha_K\}-\zeta_i(t;\beta_a)}{E\{\mathrm{dN_i(t)}=1\mid \mathbf{V}_\mathrm{i}(\mathrm{t});\hat\gamma\}}\mathrm{dN_i(t)}\right]$$
$$+pr\left[\int_0^\tau \frac{\frac{\mathbf{1}\{A_i(t)=a\}}{pr\{A_i(t)=a|\mathbf{K}_i(t);\hat\psi\}}(\mu_a\{\mathbf{V}_i(t);\hat\alpha_V\}-\mu_a\{\mathbf{K}_i(t);\hat\alpha_K\})+\mu_a\{\mathbf{K}_i(t);\hat\alpha_K\}-\zeta_i(t;\beta_a)}{E\{\mathrm{dN_i(t)}=1\mid \mathbf{V}_\mathrm{i}(\mathrm{t});\hat\gamma\}}E\{\mathrm{dN_i(t)}=1\mid \mathbf{V}_\mathrm{i}(\mathrm{t});\hat\gamma\}\right].$$

Then, using the asterisk notation for the correct models once again, we obtain

$$pr\left[\int_0^\tau \frac{\frac{\mathbf{1}\{A_i(t)=a\}}{pr\{A_i(t)=a|\mathbf{K}_i(t);\hat{\psi}\}}(Y_i(t)-\mu_a\{\mathbf{K}_i(t);\alpha_K^*\})+\mu_a\{\mathbf{K}_i(t);\alpha_K^*\}-\zeta_i(t;\beta_a)}{E\left\{\mathrm{dN_i(t)}=1\mid \mathbf{V}_\mathrm{i}(\mathrm{t});\hat{\gamma}\right\}}\mathrm{dN_i(t)}\right]$$

$$-pr\left[\int_0^\tau \frac{\frac{\mathbf{1}\{A_i(t)=a\}}{pr\{A_i(t)=a|\mathbf{K}_i(t);\hat{\psi}\}}(\mu_a\{\mathbf{V}_i(t);\alpha_V^*\}-\mu_a\{\mathbf{K}_i(t);\alpha_K^*\})+\mu_a\{\mathbf{K}_i(t);\alpha_K^*\}-\zeta_i(t;\beta_a)}{E\left\{\mathrm{dN_i(t)}=1\mid \mathbf{V}_\mathrm{i}(\mathrm{t});\hat{\gamma}\right\}}\mathrm{dN_i(t)}\right]$$

$$+pr\left[\int_0^\tau \frac{\frac{\mathbf{1}\{A_i(t)=a\}}{pr\{A_i(t)=a|\mathbf{K}_i(t);\hat{\psi}\}}(\mu_a\{\mathbf{V}_i(t);\alpha_V^*\}-\mu_a\{\mathbf{K}_i(t);\alpha_K^*\})+\mu_a\{\mathbf{K}_i(t);\alpha_K^*\}-\zeta_i(t;\beta_a)}{E\left\{\mathrm{dN_i(t)}=1\mid \mathbf{V}_\mathrm{i}(\mathrm{t});\hat{\gamma}\right\}}E\left\{\mathrm{dN_i(t)}=1\mid \mathbf{V}_\mathrm{i}(\mathrm{t});\hat{\gamma}\right\}\right]$$

$$=pr\left[\int_0^\tau \frac{\frac{\mathbf{1}\{A_i(t)=a\}}{pr\{A_i(t)=a|\mathbf{K}_i(t);\hat{\psi}\}}(Y_i(t)-\mu_a\{\mathbf{V}_i(t);\alpha_V^*\})}{E\left\{\mathrm{dN_i(t)}=1\mid \mathbf{V}_\mathrm{i}(\mathrm{t});\hat{\gamma}\right\}}\mathrm{dN_i(t)}\right] \text{ (by combining the first two lines).}$$

The last equation is unbiased since the model $\mu_a\{\mathbf{V}_i(t);\alpha_V^*\}$ is correctly specified .

In scenario c) (Table 2), we have the following equations once plugging-in the correct specified models:

$$pr\left[\int_0^\tau \frac{\frac{\mathbf{1}\{A_i(t)=a\}}{pr\{A_i(t)=a|\mathbf{K}_i(t);\hat{\psi}\}}\left[Y_i(t)-\mu_a\{\mathbf{K}_i(t);\alpha_K^*\}\right]+\mu_a\{\mathbf{K}_i(t);\alpha_K^*\}-\zeta_i(t;\beta_a)}{pr\left\{\mathrm{dN_i(t)}=1\mid \mathbf{V}_\mathrm{i}(\mathrm{t});\gamma^*\right\}}\mathrm{dN_i(t)}\right]$$

$$-pr\int_0^\tau \frac{\mathrm{dN_i(t)}-\mathrm{pr}\left\{\mathrm{dN_i(t)}=1\mid \mathbf{V}_\mathrm{i}(\mathrm{t});\gamma^*\right\}}{pr\left\{\mathrm{dN_i(t)}=1\mid \mathbf{V}_\mathrm{i}(\mathrm{t});\gamma^*\right\}}\left(\frac{\mu_a\{\mathbf{V}_i(t);\hat{\alpha}_V\}}{pr\{A_i(t)=a\mid \mathbf{K}_i(t);\hat{\psi}\}}\right)$$

$$+pr\int_0^\tau \frac{\mathrm{dN_i(t)}-\mathrm{pr}\left\{\mathrm{dN_i(t)}=1\mid \mathbf{V}_\mathrm{i}(\mathrm{t});\gamma^*\right\}}{pr\left\{\mathrm{dN_i(t)}=1\mid \mathbf{V}_\mathrm{i}(\mathrm{t});\gamma^*\right\}}\left(\frac{\mathbf{1}\{A_i(t)=a\}-pr\{A_i(t)=a\mid \mathbf{K}_i(t);\hat{\psi}\}}{pr\{A_i(t)=a\mid \mathbf{K}_i(t);\hat{\psi}\}}\mu_a\{\mathbf{K}_i(t);\alpha_K^*\}-\zeta_i(t;\beta_a)\right)$$

$$=pr\left[\int_0^\tau \frac{\mu_a\{\mathbf{K}_i(t);\alpha_K^*\}-\zeta_i(t;\beta_a)}{pr\left\{\mathrm{dN_i(t)}=1\mid \mathbf{V}_\mathrm{i}(\mathrm{t});\gamma^*\right\}}\mathrm{dN_i(t)}\right]=0.$$

The two last rows in the first development above cancel out because the martingale residuals are 0-mean under correctly specified IIV weights. The last equation above is unbiased for the causal effect since the outcome model conditional on the confounders and the IIV weights are correctly specified.

Finally, in scenario d), we transform the original equations into:

$$pr\left[\int_0^\tau \frac{\frac{\mathbf{1}\{A_i(t)=a\}}{pr\{A_i(t)=a|\mathbf{K}_i(t);\hat{\psi}\}}\left[Y_i(t)-\mu_a\{\mathbf{V}_i(t);\hat{\alpha}_V\}\right]}{E\left\{\mathrm{dN_i(t)}=1\mid \mathbf{V}_\mathrm{i}(\mathrm{t});\hat{\gamma}\right\}}\mathrm{dN_i(t)}\right]$$

$$+pr\left[\int_0^\tau\left(\frac{\mathbf{1}\{A_i(t)=a\}}{pr\{A_i(t)=a\mid \mathbf{K}_i(t);\hat{\psi}\}}\left[\mu_a\{\mathbf{V}_i(t);\hat{\alpha}_V\}-\mu_a\{\mathbf{K}_i(t);\hat{\alpha}_K\}\right]+\mu_a\{\mathbf{K}_i(t);\hat{\alpha}_K\}-\zeta_i(t;\beta_a)\right)\mathrm{dt}\right]=0.$$

Once replacing with the correctly specified models in scenario d), we obtain

$$
\begin{aligned}
& pr\left[\int_0^{\tau} \frac{\frac{\mathbf{1}\{A_i(t)=a\}}{pr\{A_i(t)=a|\mathbf{K}_i(t);\psi^*\}}\left[Y_i(t)-\mu_a\{\mathbf{V}_i(t);\alpha_V^*\}\right]}{E\left\{\mathrm{dN_i(t)}=1 \mid \mathbf{V}_\mathrm{i}(\mathrm{t});\hat{\gamma}\right\}}\mathrm{dN_i(t)}\right] \\
& + pr\left[\int_0^{\tau}\left(\frac{\mathbf{1}\{A_i(t)=a\}}{pr\{A_i(t)=a \mid \mathbf{K}_i(t);\psi^*\}}\left[\mu_a\{\mathbf{V}_i(t);\alpha_V^*\}-\mu_a\{\mathbf{K}_i(t);\hat{\alpha}_K\}\right]+\mu_a\{\mathbf{K}_i(t);\hat{\alpha}_K\}-\zeta_i(t;\beta_a)\right)\mathrm{dt}\right] \\
& = pr\left[\int_0^{\tau} \frac{\frac{\mathbf{1}\{A_i(t)=a\}}{pr\{A_i(t)=a|\mathbf{K}_i(t);\psi^*\}}\left[Y_i(t)-\mu_a\{\mathbf{V}_i(t);\alpha_V^*\}\right]}{E\left\{\mathrm{dN_i(t)}=1 \mid \mathbf{V}_\mathrm{i}(\mathrm{t});\hat{\gamma}\right\}}\mathrm{dN_i(t)}\right] \\
& + pr\left[\int_0^{\tau}\left(\frac{\mathbf{1}\{A_i(t)=a\}}{pr\{A_i(t)=a \mid \mathbf{K}_i(t);\psi^*\}}\mu_a\{\mathbf{V}_i(t);\alpha_V^*\}\right)dt\right] \\
& - pr\left[\left(\frac{\mathbf{1}\{A_i(t)=a\}-pr\{A_i(t)=a \mid \mathbf{K}_i(t);\psi^*\}}{pr\{A_i(t)=a \mid \mathbf{K}_i(t);\psi^*\}}\mu_a\{\mathbf{K}_i(t);\hat{\alpha}_K\}+\zeta_i(t;\beta_a)\right)\mathrm{dt}\right] \\
& = pr\left[\int_0^{\tau}\left(\frac{\mathbf{1}\{A_i(t)=a\}}{pr\{A_i(t)=a \mid \mathbf{K}_i(t);\psi^*\}}\mu_a\{\mathbf{V}_i(t);\alpha_V^*\}-\zeta_i(t;\beta_a)\right)dt\right]=0.
\end{aligned}
$$

The last equation above is unbiased for the causal effect.

Web Appendix C: Relation with model-assisted estimation

The AAIIW estimator uses the theory introduced in Robins et al. (1994), well laid out in e.g., Funk et al. (2011), and that can be related to model-assisted estimation from the survey sampling field, see e.g., the discussion in Chambers (1998).

Using the model-assisted estimation approach to justify the construction of the novel estimator may be more intuitive to some readers, compared with using the semiparametric theory of influence functions. Related discussions can also be found in Jiang et al. (2022). We briefly discuss that framework in this Appendix.

The estimating equations of the FIPTM estimator are to be transformed twice following the model-assisted estimation approach. Based on Robins et al. (1994), Robins and Rotnitzky in the paper of Chambers (1998) introduce the equation:

$$N\hat{T}_{diff}(\mu) = \sum_{i=1}^{N} A_i \mu(\mathbf{X}_i) + \sum_{i:A_i=a} A_i \left\{ Y_i - \mu(\mathbf{X}_i) \right\} / \pi_i$$

and connect it to model-assisted estimation, where $\hat{T}_{diff}(\mu)$ is a designed-based standard difference estimator for the parameter $E[A_i Y_i]$ of interest, and $\mu(\mathbf{X})$ is a function of $\mathbf{X}$. Theorem 1 in chambers (1998) implies that the class of such estimators $\hat{T}_{diff}(\mu)$ contains all the semiparametric estimators, and that the asymptotic variance of the estimator evaluated at $\mu_{eff}(x) = E[Y_i \mid \mathbf{X}_i = \mathbf{x}]$ in their notation leads to the smallest variance possible.

We use that construction twice, starting first with $A_i = \mathbf{1}\{A_i(t) = a\}$, $\pi_i = pr\{A_i(t) = a \mid \mathbf{K}_i(t)\}$ and "$\mu(\mathbf{X}_i)$" $= E\left[Y_i(t) \mid A_i(t) = a, \mathbf{K}_i(t)\right]$ using their notation. Once this projection is obtained, we use the strategy a second time, with the "Y_i" now corresponding to the previous expression obtained, and with "A_i" $= \mathrm{dN_i(t)}$, "π_i" $= pr\{\mathrm{dN_i(t)} = 1 \mid \mathrm{V_i(t)}\}$ and a novel "$\mu(\mathbf{X}_i)$" function corresponding to the expectation of the previous term before augmentation (this expectation corresponds to $E[\eta_i(t) \mid A_i(t) = a, \mathbf{K}_i(t), \mathbf{V}_i(t)]$ in the estimating equations of the AAIIW presented in the main manuscript).

By construction, the novel proposed estimator is the most efficient among its class of semiparametric estimators, which also includes the FIPTM estimator. The correspondence between the model-based estimation approach and the geometry of influence functions can be explained by noting that the novel estimator's influence function corresponds to sequential projections of the FIPTM's influence function onto spaces orthogonal to the residuals from the weight models and orthogonal to projections due to the outcome mean models onto the spaces of $\mathbf{K}_i(t)$ and $\mathbf{V}_i(t)$.

References

Chambers, R. L. (1998). Discussion on the papers by Firth and Bennett and Pfeffermann et al. Journal of the Royal Statistical Society, Series B (Statistical Methodology), 60(1), pp. 51-52.

Funk, M. J., Westreich, D., Wiesen, C., Stürmer, T., Brookhart, M. A., & Davidian, M. (2011). Doubly robust estimation of causal effects. American journal of epidemiology, 173(7), pp. 761-767.

Jiang, Z., Yang, S., & Ding, P. (2022). Multiply robust estimation of causal effects under principal ignorability. Journal of the Royal Statistical Society Series B: Statistical Methodology, 84(4), pp. 1423-1445.

Robins, J. M., Rotnitzky, A., & Zhao, L. P. (1994). Estimation of regression coefficients when some regressors are not always observed. Journal of the American statistical Association, 89(427), pp. 846-866.

Web Appendix D: Meaning of a correct specification of a model

Correct specification for a nuisance model requires that the corresponding data generating mechanism (DGM) can be modelled parametrically and that there exists a true set of parameters leading to the actual DGM that we can estimate consistently (we denote the true sets by $\boldsymbol{\psi}_0$, $\boldsymbol{\gamma}_0$, $\boldsymbol{\alpha}_{K0}$ and $\boldsymbol{\alpha}_{V0}$ for the treatment, observation, and two conditional mean outcome models, respectively).

For instance, the treatment model being correctly specified implies that the DGM $pr\{A_i(t) = a \mid \mathbf{K}_i(t)\} = pr\{A_i(t) = a \mid \mathbf{K}_i(t); \boldsymbol{\psi}_0\}$, that we can model this DGM using the correct functional formats for covariates $\mathbf{K}_i(t)$ in the model, and that estimators $\hat{\boldsymbol{\psi}}$ converge in probability to the true parameters $\boldsymbol{\psi}_0$.

Web Appendix E: Relative asymptotic efficiency of AAIIW

To make the demonstrations lighter, denote

$$\begin{aligned} I_a =& \mathbf{1}\{A_i(t) = a\} \\ e_a =& pr\{A_i(t) = a \mid \mathbf{K}_i(t); \psi_0\} \\ \mathrm{dN} =& \mathbf{1}\{\mathrm{dN_i(t)} = 1\} \\ \rho =& E\{\mathrm{dN_i(t)} = 1 \mid \mathbf{V}_\mathrm{i}(\mathrm{t}); \gamma_0\} \\ \mathrm{dM} =& \mathrm{dN_i(t)} - \rho \\ \mu_{aK} =& \mu_a\{\mathbf{K}_i(t); \alpha_{0K}\} \\ \mu_{aV} =& \mu_a\{\mathbf{V}_i(t); \alpha_{0V}\} \\ \mu_0 =& E[Y_i^0(t)] = \beta_0 \\ \mu_1 =& E[Y_i^1(t)] = \beta_0 + \beta_1. \end{aligned}$$

With the simpler notation, and under all nuisance models correctly specified, the FIPTM (in this case, we do not consider modelling flexibly the intercept as in the original paper proposing the FIPTM but rather consider a standard doubly weighted least squares estimator for the FIPTM) and AAIIW estimators respectively correspond to the solutions of the following sets of estimating equations:

$$E_n \left[\int_0^\tau \frac{\mathrm{dN}}{\rho} \frac{I_a}{e_a} \{Y_i(t) - \zeta_i(t; \beta_a)\} \right] = 0 \tag{1}$$

and

$$E_n \left[\int_0^\tau \left\{ \frac{I_a}{e_a} Y_i(t) - \left(\frac{I_a - e_a}{e_a} \right) \mu_{aK} - \zeta_i(t; \beta_a) \right\} \frac{\mathrm{dN}}{\rho} \right] - E_n \left[\int_0^\tau \frac{\mathrm{dM}}{\rho} \left\{ \frac{\mu_{aV}}{e_a} - \left(\frac{I_a - e_a}{e_a} \right) \mu_{aK} - \zeta_i(t; \beta_a) \right\} \right] = 0. \tag{2}$$

Note that each set of estimating equations contains two equations, one for β_0 and one for β_1 once β_0 is evaluated. We use the influence function of each estimator to derive its asymptotic variance. The variance of each estimator equals to the variance of its influence function.

Denote by $\beta_a = [\beta_0 \ \beta_1]$ the vector of true parameters. We prove the relative efficiency of the AAIIW estimator when compared with the FIPTM estimator under the assumption that all nuisance models are correctly specified for both estimators. The influence function of the $\hat{\beta}_{FIPTM}$ estimator, denoted $\psi(o_i) = \psi(o_i; \beta_a, \delta_0)$ for o_i the observation data of the ith individual, and $\delta_0 = \{\psi_0, \gamma_0\}$ the true nuisance parameters (for the treatment and observation models) must satisfy

$$\sqrt{n} \left(\hat{\beta}_{FIPTM} - \beta_a \right) = \frac{1}{\sqrt{n}} \sum_{i=1}^n \psi(o_i) + \mathcal{O}(1).$$

By replacing terms in equation 1 above, we obtain a vector of influence functions the same size as the vector of parameters of interest β_a, that is:

$$\psi(o_i) = \begin{bmatrix} \int_{t=0}^\tau \frac{\mathrm{dN}}{\rho} \frac{I_0}{e_0} \{Y_i(t) - \mu_0\} \\ \int_{t=0}^\tau \frac{\mathrm{dN}}{\rho} \left\{ \left(\frac{I_1}{e_1} - \frac{I_0}{e_0} \right) Y_i(t) + \mu_0 I_0 - \mu_1 I_1 \right\} \end{bmatrix}.$$

For the variance of the FIPTM estimator, our interest is in β_1 and so we focus on the second equation (the only equation that depends on it), assuming we already have an estimate for β_0 from the first equation. Using similar developments as those that follow, we could show that the variance of the estimate for β_0 is also smaller when using the AAIIW estimator as compared with the FIPTM estimator, but this is omitted in what follows.

The variance of the FIPTM estimator equals to the variance of its influence function. We denote that variance by σ^2_{FIPTM}. In further derivations, we drop the integral that sums the terms over all times when there is an observation of the outcome (as this integral sum is the same for the FIPTM and the AAIIW estimator). We, therefore, focus on only one term in the integral, yielding:

$$
\begin{aligned}
\sigma^2_{FIPTM} =& E[\psi(o_i)^2] + E[\psi(o_i)]^2 \\
=& E[\psi(o_i)^2] + 0 \\
=& E\left(\left[\frac{\mathrm{dN}}{\rho}\left\{\left(\frac{I_1}{e_1}-\frac{I_0}{e_0}\right)Y_i(t)+\mu_0 I_0-\mu_1 I_1\right\}\right]^2\right) \\
=& E\left[\overbrace{\left\{\frac{\mathrm{dN}}{\rho}\left(\frac{I_1}{e_1}\right)(Y_i(t)-\mu_1)\right\}}^{A}{}^2\right] + E\left[\overbrace{\left\{\frac{\mathrm{dN}}{\rho}\left(\frac{I_0}{e_0}\right)(Y_i(t)-\mu_0)\right\}}^{B}{}^2\right] - 2E\left[E\left\{A\times B \mid \mathbf{V}_i(t)\right\}\right] \\
=& E\left[\frac{\mathrm{dN}}{\rho^2}\frac{I_1}{e_1^2}(Y_i(t)-\mu_1)^2\right] + E\left[\frac{\mathrm{dN}}{\rho^2}\frac{I_0}{e_0^2}(Y_i(t)-\mu_0)^2\right] - 2E\left\{E\left[A \mid \mathbf{V}_i(t)\right]\times E\left[B \mid \mathbf{V}_i(t)\right]\right\}
\end{aligned}
$$

with the third term true since both terms are independent given V (treated and untreated patients). Now, the third term is 0 because each expectation conditional on $\mathbf{V}_i(t)$ is zero.

$$
\begin{aligned}
&= E\left[\frac{E[\mathrm{dN} \mid \mathbf{V}_\mathrm{i}(\mathrm{t})]}{\rho^2}\frac{E[I_1 \mid \mathbf{V}_i(t)]}{e_1^2}E\left[(Y_i(t)-\mu_1)^2 \mid \mathbf{V}_i(t), A_i(t)=1, \mathrm{dN_i(t)}=1\right]\right] \\
&\quad + E\left[\frac{E[\mathrm{dN} \mid \mathbf{V}_\mathrm{i}(\mathrm{t})]}{\rho^2}\frac{E[I_0 \mid \mathbf{V}_i(t)]}{e_0^2}E\left[(Y_i(t)-\mu_0)^2 \mid \mathbf{V}_i(t), A_i(t)=0, \mathrm{dN_i(t)}=1\right]\right] + 0 \\
&= E\left[\frac{1}{\rho}\frac{1}{e_1}E\left[(Y_i(t)-\mu_1)^2 \mid \mathbf{V}_i(t), A_i(t)=1, \mathrm{dN_i(t)}=1\right]\right] \\
&\quad + E\left[\frac{1}{\rho}\frac{1}{e_0}E\left[(Y_i(t)-\mu_0)^2 \mid \mathbf{V}_i(t), A_i(t)=0, \mathrm{dN_i(t)}=1\right]\right] \\
&= E\left[\frac{(Y_i^1(t)-\mu_1)^2}{\rho e_1}\right] + E\left[\frac{(Y_i^0(t)-\mu_0)^2}{\rho e_0}\right]. \qquad (3)
\end{aligned}
$$

For the AAIIW estimator, we need to show that the variance is smaller than (3). First we derive the influence function $\phi(o_i)=\phi(o_i;\beta_a,\delta_0)$ for o_i the observation data of the ith individual and δ_0 now defined as the vector of true parameters $\{\psi_0,\gamma_0,\alpha_{K0},\alpha_{V0}\}$ (which now includes the parameters of the two conditional outcome mean models). We have that the influence function satisfies

$$
\sqrt{n}(\hat{\beta}_{AAIIW}-\beta_a)=\frac{1}{\sqrt{n}}\sum_{i=1}^{n}\phi(o_i)+o(1)
$$

or, using the formula for M-estimators provided in Tsiatis (Chapter 3, section 3.2) and noting that the AAIIW estimator is an M-estimator and is the solution to

$$
\sum_{i=1}^{n} m(o_i;\hat{\beta}_{AAIIW})=0 \qquad (4)
$$

with the m-function that can be derived directly from (4), then we can compute the influence function by first computing the partial derivatives of the m function w. r. t. to the two parameters in β_a (use, e.g., formula 3.6 in Tsiatis) and find that

$$
\phi(o_i) = \psi(o_i) + \begin{bmatrix}
\int_{t=0}^{\tau} -\frac{\mathrm{dN}}{\rho}\left(\frac{I_0-e_0}{e_0}\right)\mu_{0K} - \frac{\mathrm{dM}}{\rho}\left\{\frac{\mu_{0V}}{e_0}-\left(\frac{I_0-e_0}{e_0}\right)\mu_{0K}-\mu_0\right\} \\
\int_{t=0}^{\tau} -\frac{\mathrm{dN}}{\rho}\left(\frac{I_1-e_1}{e_1}\right)\mu_{1K} - \frac{\mathrm{dM}}{\rho}\left\{\frac{\mu_{1V}}{e_1}-\left(\frac{I_1-e_1}{e_1}\right)\mu_{1K}-\mu_1\right\} \\
+\frac{\mathrm{dN}}{\rho}\left(\frac{I_0-e_0}{e_0}\right)\mu_{0K} + \frac{\mathrm{dM}}{\rho}\left\{\frac{\mu_{0V}}{e_0}-\left(\frac{I_0-e_0}{e_0}\right)\mu_{0K}-\mu_0\right\}
\end{bmatrix}
$$

with the second part added to $\psi(o_i)$ corresponding to the augmented terms. Note that the second row of $\phi(o_i)$ contains two terms, one being the augmented term for the treated and one for the untreated. The change in sign for both terms is due to the subtraction between the augmented terms due to the treated and the untreated patients.

To compute the variance of the AAIIW, that we denote by σ^2_{AAIIW}, we compute the variance of its influence function. We again focus on the second influence function assuming that β_0 is already estimated, and again we drop the integral sign in the following derivations. We have:

$$\begin{aligned}
\sigma^2_{AAIIW} =& E[\phi(o_i)^2] \\
=& E\left[\psi(o_i)^2\right] \\
&+ E\left[\left(\overbrace{-\left\{\frac{I_1 - e_1}{e_1}\right\}\mu_{1K} - \frac{\text{dM}}{\rho}\left\{\frac{\mu_{1V}}{e_1} - \mu_1\right\}}^{C_1} + \overbrace{\left\{\frac{I_0 - e_0}{e_0}\right\}\mu_{0K} + \frac{\text{dM}}{\rho}\left\{\frac{\mu_{0V}}{e_0} - \mu_0\right\}}^{C_2}\right)^2\right] \\
&+ 2E[\psi(o_i) \times (C_1 + C_2)].
\end{aligned}$$

The first term, $E\left[\psi(o_i)^2\right]$, is the variance of the FIPTM estimator. Denote the second and third terms by $D = E[(C_1 + C_2)^2]$ and $F = 2E[\psi(o_i) \times (C_1 + C_2)]$. We must show that $D + F < 0$ to show relative efficiency of the AAIIW estimator. We have that

$$D = E[(C_1 + C_2)^2] = E[C_1^2 + C_2^2 + 2C_1C_2] = E[C_1^2] + E[C_2^2] + E[2C_1C_2]$$

where

$$\begin{aligned}
E[C_1^2] =& E\left[\frac{\text{dM}^2}{\rho^2}\left(\frac{\mu_{1V}}{e_1} - \mu_1\right)^2\right] + E\left[\left(\frac{I_1 - e_1}{e_1}\right)^2 \mu_{1K}^2\right] \\
&- 2E\left[E\left[-\frac{\text{dM}}{\rho}\left\{\frac{\mu_{1V}}{e_1} - \mu_1\right\}\left(\frac{I_1 - e_1}{e_1}\right)\mu_{1K} | \mathbf{V}_i(t)\right]\right] \\
=& E\left[\frac{\text{dM}^2}{\rho^2}\left(\frac{\mu_{1V}}{e_1} - \mu_1\right)^2\right] + E\left[\frac{I_1 + e_1^2 - 2I_1e_1}{e_1^2}\mu_{1K}^2\right] \\
&\text{where the third term was removed since } E\left[\text{dM}| \mathbf{V}_\text{i}(\text{t})\right] = 0 \\
=& E\left[\frac{E[(\text{dN} + \rho^2 - 2\text{dN}\rho) \mid \mathbf{V}_\text{i}(\text{t})]}{\rho^2}\left(E[Y_i^1(t)] - \mu_1\right)^2\right] + \mu_1^2 E\left[\frac{E\left[I_1 + e_1^2 - 2I_1e_1 \mid \mathbf{V}_i(t)\right]}{e_1^2}\right] \\
=& 0 + \mu_1^2 E\left[\frac{1 - e_1}{e_1}\right] \quad \text{since } E[Y_i^1(t)] - \mu_1 = 0.
\end{aligned}$$

Similarly, we have

$$E[C_2^2] = \mu_0^2 E\left[\frac{1 - e_0}{e_0}\right].$$

The third term, $E[2C_1C_2]$, cancels out, after using iterated expectation and conditioning on e.g., $\mathbf{V}_i(t)$, as the martingale residuals and the treatment residuals are zero-mean conditional on that set. Thus,

$$D = \mu_1^2 E\left[\frac{1 - e_1}{e_1}\right] + \mu_0^2 E\left[\frac{1 - e_0}{e_0}\right].$$

We are left with calculting F. We have:

$$\begin{aligned}
F =& 2E[\psi(o_i) \times (C_1 + C_2)] \\
=& 2E[\psi(o_i) \times C_1] + 2E[\psi(o_i) \times C_2].
\end{aligned}$$

We start with the left term and have

$$
\begin{aligned}
E[\psi(o_i)\times C_1] =& E\left[\frac{\mathrm{dN}}{\rho}\left\{\left(\frac{I_1}{e_1}-\frac{I_0}{e_0}\right)Y_i(t)+\mu_0 I_0-\mu_1 I_1\right\}\times\left(-\left\{\frac{I_1-e_1}{e_1}\right\}\mu_{1K}-\frac{\mathrm{dM}}{\rho}\left\{\frac{\mu_{1V}}{e_1}-\mu_1\right\}\right)\right]\\
=& -E\left[\frac{\mathrm{dN}}{\rho}\left\{\left(\frac{I_1}{e_1}-\frac{I_0}{e_0}\right)Y_i(t)+\mu_0 I_0-\mu_1 I_1\right\}\times\left(\left\{\frac{I_1-e_1}{e_1}\right\}\mu_{1K}+\frac{\mathrm{dM}}{\rho}\left\{\frac{\mu_{1V}}{e_1}-\mu_1\right\}\right)\right]\\
=& -E\left[\frac{\mathrm{dN}}{\rho}\left\{\frac{I_1}{e_1}Y_i(t)-\mu_1\right\}\times\left\{\frac{I_1-e_1}{e_1}\right\}\mu_{1K}\right]+E\left[\frac{\mathrm{dN}}{\rho}\left\{\frac{I_0}{e_0}Y_i(t)-\mu_0\right\}\times\left\{\frac{I_1-e_1}{e_1}\right\}\mu_{1K}\right]\\
&-E\left[\frac{\mathrm{dN}}{\rho}\left\{\frac{I_1}{e_1}Y_i(t)-\mu_1\right\}\times\frac{\mathrm{dM}}{\rho}\left\{\frac{\mu_{1V}}{e_1}-\mu_1\right\}\right]+E\left[\frac{\mathrm{dN}}{\rho}\left\{\frac{I_0}{e_0}Y_i(t)-\mu_0\right\}\times\frac{\mathrm{dM}}{\rho}\left\{\frac{\mu_{1V}}{e_1}-\mu_1\right\}\right]\\
=& -E\left[\frac{\mathrm{dN}}{\rho}\left\{\frac{I_1-e_1I_1}{e_1^2}Y_i(t)-\left\{\frac{I_1-e_1}{e_1}\right\}\mu_1\right\}\times\mu_{1K}\right]\\
&+E\left[\frac{\mathrm{dN}}{\rho}\left\{\frac{I_0(I_1-e_1)}{e_0e_1}Y_i(t)-\left\{\frac{I_1-e_1}{e_1}\right\}\mu_0\right\}\times\mu_{1K}\right]\\
&-E\left[\frac{\mathrm{dN}-\mathrm{dN}\rho}{\rho^2}\left\{\frac{I_1}{e_1}Y_i(t)-\mu_1\right\}\times\left\{\frac{\mu_{1V}}{e_1}-\mu_1\right\}\right]\\
&+E\left[\frac{\mathrm{dN}-\mathrm{dN}\rho}{\rho^2}\left\{\frac{I_0}{e_0}Y_i(t)-\mu_0\right\}\times\left\{\frac{\mu_{1V}}{e_1}-\mu_1\right\}\right].
\end{aligned}
$$

Taking iterated expectation and conditioning on $\mathbf{V}_i(t)$, several residuals cancel out and we are left with

$$
\begin{aligned}
&= -E\left[\frac{1-e_1}{e_1}\mu_1^2\right]-E\left[\mu_1^2\right]-0+0\\
&= -\mu_1^2 E\left[\frac{1}{e_1}\right]
\end{aligned}
$$

Similarly, we find

$$E[\psi(o_i)\times C_2] = -\mu_0^2 E\left[\frac{1}{e_0}\right].$$

The variance of the AAIIW is therefore obtained by summing:

$$
\begin{aligned}
\sigma^2_{AAIIW} &= \sigma^2_{FIPTM}+D+F\\
&= \sigma^2_{FIPTM}+\mu_1^2E\left[\frac{1-e_1}{e_1}\right]+\mu_0^2E\left[\frac{1-e_0}{e_0}\right]-2\mu_1^2E\left[\frac{1}{e_1}\right]-2\mu_0^2E\left[\frac{1}{e_0}\right]\\
&\iff\\
\sigma^2_{AAIIW}-\sigma^2_{FIPTM} &= \mu_1^2E\left[\frac{1-e_1}{e_1}-\frac{2}{e_1}\right]+\mu_0^2E\left[\frac{1-e_0}{e_0}-\frac{2}{e_0}\right].
\end{aligned}
$$

And, since $\mu_1^2, \mu_0^2 > 0$, and

$$\frac{1-e_1}{e_1}-\frac{2}{e_1}=\frac{-1-e_1}{e_1}\leq\frac{-1}{e_1}<0 \text{ for all } e_1 \text{ s. t. } 0\leq e_1\leq 1$$

and

$$\frac{1-e_0}{e_0}-\frac{2}{e_0}=\frac{-1-e_0}{e_0}\leq\frac{-1}{e_0}<0 \text{ for all } e_0 \text{ s. t. } 0\leq e_0\leq 1$$

we find that

$$
\begin{aligned}
&\sigma^2_{AAIIW}-\sigma^2_{FIPTM}<0\\
\iff &\sigma^2_{AAIIW}<\sigma^2_{FIPTM}
\end{aligned}
$$

under all nuisance models correctly specified for both estimators. Thus, the FIPTM asymptotic variance equals to

$$\sigma^2_{FIPTM}=E\left[\frac{\{Y_i^1(t)-\mu_1\}^2}{\rho\{\mathbf{V}_i(t)\}e_1\{\mathbf{K}_i(t)\}}\right]+E\left[\frac{\{Y_i^0(t)-\mu_0\}^2}{\rho\{\mathbf{V}_i(t)\}e_0\{\mathbf{K}_i(t)\}}\right],$$

where $\mu_a = E[Y_i^a(t)]$, $\rho\{\mathbf{V}_i(t)\} = E[\mathrm{dN_i(t)} \mid \mathbf{V}_\mathrm{i}(\mathrm{t}); \gamma_0]$, and $e_a\{\mathbf{K}_i(t)\} = pr\{A_i(t) = a \mid \mathbf{K}_i(t); \psi_0\}$. The augmented AAIIW estimator is more efficient asymptotically, with asymptotic variance

$$\sigma^2_{AAIIW} = \sigma^2_{FIPTM} - \mu_1^2 E\left[\frac{1 + e_1\{\mathbf{K}_i(t)\}}{e_1\{\mathbf{K}_i(t)\}}\right] - \mu_0^2 E\left[\frac{1 + e_0\{\mathbf{K}_i(t)\}}{e_0\{\mathbf{K}_i(t)\}}\right].$$

References

Tsiatis, A. A. (2006). Semiparametric theory and missing data. Springer: New York.

Web Appendix F: Addressing informative censoring with the multiply robust estimator

Although we are in a repeated measures setting as opposed to a survival setting, different patients may have different censoring times associated with measured characteristics that are also related to the outcome, the exposure, or both. An adjustment for informative censoring may be necessary to avoid biased estimates. Inverse probability of censoring weights (IPCW) (Rotnitzky and Robins, 2005) can be incorporated in the AAIIW estimating equations to address this additional challenge. These weights, if they are correctly specified, create a pseudo-population in which the missing follow-up time of patients who were censored is artificially recovered by upweighting similar patients with non-censored follow-up times.

An additional challenge with informative censoring that comes up with the more naive IPT-weighted or FIPTM estimator and the AAIIW is that the propensity score model and the outcome mean model conditioning on the confounders will be fitted on the non-censored observations only. For instance, the propensity score we estimate in non-censored data is an estimate of $P(A_i(t) = 1 \mid \mathbf{K}_i(t), \xi_i(t) = 1)$ while we are interested in the PS $P(A_i(t) = 1 \mid \mathbf{K}_i(t))$. To address this challenge, one has to fit the PS and the outcome mean model conditional on the confounders, $\mu_a\{\mathbf{K}_i(t); \boldsymbol{\alpha}_K\}$, in an IPC-weighted pseudo-population. This is discussed more in Section 2 below. In Section 3 below, we also outline how one could develop an extension of our multiply robust estimator that is even more robust, that considers a censoring model (like the one used in IPCW) and a conditional outcome model that conditions on censoring predictors.

1. Computing the cumulated inverse probability of censoring weights

The probability of still being in the study at time t, denoted $P(\xi_i(t) = 1)$, depends on the follow-up and evolving characteristics up to time t. The IPCW is a cumulated inverse probability of censoring weight and it should be adjusted for all the evolving covariates during follow-up that led to additional spurious dependence between the exposure and the outcome after conditioning on the respective censoring indicators, for any time t. This involves cumulating the probability of not being censored at each time $s = 0, ..., t$. Assuming that censoring depends on the same set of visit predictors, $\mathbf{V}_i(t)$, as the observation of the outcome, we suggest the users of the multiply robust estimator to use inverse probability of censoring weights (IPCW) given by the inverse of the following fitted joint probability at time t. The IPC weight at time t is given by the inverse of:

$$P(\xi_i(t) = 1 \mid \mathcal{H}_i(t)) = \prod_{s=0}^{t} P(\xi_i(s) = 1 \mid \mathbf{V}_i(s)),$$

where $\mathcal{H}_i(t)$ denotes the full covariates history, and assuming sequential exchangeability as described below. Cumulating the weight ensures that, at time t, after IPC-weighting, any paths that could relate $A(t)$ and $Y(t)$ spuriously, that is due to censoring, (including paths due to any previous covariates accounted for in the censoring model) are blocked.

These weights are appropriate to adjust for informative censoring if the conditional independence assumption $A_i(t) \perp \xi_i(t) \mid \mathcal{H}_i(t)$ is met.

We also outline the need to stabilize the IPCW. These weights can be highly variable since they are cumulated over several time points (e.g., 730 time points, for a two-year follow-up when information could be collected daily). To stabilize the weights, one can fit a similar model for censoring as $P(\xi_i(s) = 1 \mid \mathbf{V}_i(s))$ but using only a constant and no predictor, or one as a function of baseline variables (e.g., baseline confounders). Then, the stabilizer can be incorporated as the numerator of the weight which is further cumulated over time points up to t.

2. Adapting the propensity score for informative censoring

Both the new proposed approach and the previous, doubly-weighted estimator, use a treatment model (so-called propensity score) in their estimating equations. In addition, the new AAIIW estimator uses an outcome mean model conditioning on confounders denoted by $\mathbf{K}_i(t)$.

The PS (treatment model) is denoted by $P(A_i(t) = 1 \mid \mathbf{K}_i(t))$. In observed data, one can only fit the model $P(A_i(t) = 1 \mid \mathbf{K}_i(t), \xi_i(t) = 1; \hat{\boldsymbol{\omega}}) = E(\xi_i(t)A_i(t) \mid \mathbf{K}_i(t); \hat{\boldsymbol{\omega}})$ when there is censoring and

data are not observed after a certain time point. We assume the following conditional independence assumption

$$A_i(t) \perp \xi_i(t) \mid \mathcal{H}_i(t)$$

and also assume sequential conditional exchangeability, i.e., $\xi_i(t-1) \perp \xi_i(t) \mid \mathbf{V}_i(t)$ (meaning that at time t, only the visit predictors at that time are needed to block additional spurious association due to that time). We can fit the propensity score and the outcome mean model conditional on the confounders in an IPC-weighted pseudo-population. We only show the consistency of the IPC-weighted estimation of the PS below (and let the derivation for the outcome mean model $\mu_a\{\mathbf{K}_i(t)\}$ to the interested reader).

Below, we removed the time index and the bold notation for matrices or vectors, for simplicity, and we show how the IPC-weighted propensity score is consistent for the PS we are interested in. We start by showing the estimating equations we would be using in the observed (non-censored) data, if we used a logistic regression model for the exposure:

$$\begin{aligned}
E\left[\left(A\xi - \frac{\exp(\omega K)}{1+\exp(\omega K)}\right)K\right] &= E_{\mathcal{H}}\left[E\left[\left(A\xi - \frac{\exp(\omega K)}{1+\exp(\omega K)}\right)K\middle|\mathcal{H}\right]\right] \\
&= E_{\mathcal{H}}\left[K\cdot E\left[\left(A\xi - \frac{\exp(\omega K)}{1+\exp(\omega K)}\right)\middle|\mathcal{H}\right]\right] \\
&= E_{\mathcal{H}}\left[K\cdot\left\{E\left[A\xi|\,\mathcal{H}\right] - E\left[\frac{\exp(\omega K)}{1+\exp(\omega K)}\middle|\mathcal{H}\right]\right\}\right] \\
&= E_{\mathcal{H}}\left[K\cdot\left\{E\left[A|\,\mathcal{H}\right]E\left[\xi|\,\mathcal{H}\right] - \frac{\exp(\omega K)}{1+\exp(\omega K)}\right\}\right] \\
&= E_{\mathcal{H}}\left[K\cdot\left\{\frac{\exp(\omega K)}{1+\exp(\omega K)}E\left[\xi|\,\mathcal{H}\right] - \frac{\exp(\omega K)}{1+\exp(\omega K)}\right\}\right].
\end{aligned}$$

To obtain unbiased estimating equations (i.e., the equations above to be of null expectation), we must start with a weighted exposure A, i.e., $A/E[\xi \mid \mathcal{H}]$, so that the expectation $E[\xi \mid \mathcal{H}]$ in the left part in the equation above cancels out and the subtraction equals zero. Thus, by incorporating IPCW in, e.g., the logistic regression model for the treatment, we obtain consistent estimates of the PS corresponding to a cohort with no informative censoring.

3. Proposal for extending the multiply robust estimator for censoring by augmenting the current estimating equations

For the reader interested in extending the proposed multiply robust to be robust with respect to the censoring model, in addition to the visit and the treatment models, we outline below a potential extension of the estimator that is inspired by the augmented inverse probability weighted estimator proposed by Rotnitzky and Robins (2005).

Denote by $U_{1i}(\beta)$ the ith estimating equation for the average causal effect proposed in the main manuscript, i.e., the AAIIW equations. An extension that is more robust to misspecification of the censoring model used in the IPC weights is based on an augmented residual term for the censoring model. The new equations would be given by

$$U_{2i}(\beta) = \frac{U_{1i}(\beta)\xi_i(t)}{P\{\xi_i(t) \mid \mathcal{H}_i(t)\}} - \int_{s=0}^{\tau}\left[\frac{\xi_i(t) - P\{\xi_i(t) \mid \mathcal{H}_i(t)\}}{P\{\xi_i(t) \mid \mathcal{H}_i(t)\}}\right]E\left[U_{i1}(\beta) \mid \mathcal{H}_i(t)\right].$$

Derivation of the theoretical properties of that estimator are left to the reader and can be based on the reference below.

References

Rotnitzky, A., and Robins, J. (2005). *Inverse probability weighted estimation in survival analysis.* Encyclopedia of Biostatistics, vol. 4, pp. 2619-2625.

Web Appendix G: Additional details on the simulation setup

Simulation studies details

Data were simulated to emulate a setting in which time is continuous and covariates can be measured or updated at any point in time. For that, we used a time grid starting at time 0 spanning up to time $\tau = 2$ over which the variables were simulated at each 0.1-width time bin. First, the confounders, the treatment, the mediator, the pure predictor and the outcome were simulated over each of these small time bins, after which the observation process was simulated (and outcomes were removed). The individual index is omitted in what follows.

For each individual, three baseline confounders were simulated as $K_1 \sim N(1, 1)$, $K_2 \sim$ Bernoulli(0.55) and $K_3 \sim N(0, 1)$ and were repeated throughout follow-up time. At each time t, a time-varying binary treatment was simulated as $A(t) \sim$ Bernoulli(p_t) with $p_t = \text{expit}(-0.5 + 0.8\ K_1 - 0.4\ K_2 - 0.4\ K_3)$ where $\text{expit}(j) = \exp(j)/\{1 + \exp(j)\}$ and the confounders are kept time-fixed (i.e., we emulate a setting in which they are measured at baseline). At each time t, a mediator $M(t)$ of the causal effect of $A(t)$ on the outcome $Y(t)$ was further simulated as $M(t) \mid A(t) = 1 \sim N(2, 1)$ and $M(t) \mid A(t) = 0 \sim N(4, 2)$ where the parameters in the Normal respectively correspond to the mean and variance of the random variable. A time-varying pure predictor was simulated as $P(t) \sim N(0.5, 0.09)$.

A few details are particularly important in the simulation of the outcome $Y(t)$. First, since we would like to assess the performance of the different approaches when a mediator affects the outcome observation process, the outcome must depend on the mediator $M(t)$ and the mediator must affect the outcome observation. However, we would like to know the gold standard for the causal effect in the simulations, i.e., the true causal marginal effect of treatment once we marginalize the outcome mean model over $M(t)$. For that, we first modeled $E[M(t) \mid A(t), K_1, K_2, K_3]$ using a least squares linear model. Then, we simulated the outcome as

$$Y(t) = \kappa + 1\ A(t) + 0.4\ K_1 + 0.05\ K_2 - 0.6\ K_3 +$$

$$3\{M(t) - E[M(t) \mid A(t), K_1, K_2, K_3]\} + 0.3\ P(t) + \epsilon(t)$$

where $\kappa = 0.5$, $\epsilon(t) \sim N(\phi, 0.01)$, and $\phi \sim N(0, 0.04)$. We let ϕ vary by individual. When marginalizing over $M(t)$, we obtain a true marginal causal effect of 1 for treatment $A(t)$. This would not be the case if we had interactions between the treatment $A(t)$ and the confounders K in the outcome generating mechanism above.

The outcome above was first simulated once for each point on the grid. The outcome values were then set to missing according to a covariate-dependent observation process with an observation rate (Poisson model) or probability (Bernoulli model) depending on the treatment, the mediator, the pure predictor, and the confounders. For each time point, this was done by simulating an observation indicator (one minus a missingness indicator). In a first set of simulation studies, we used a non-homogeneous Poisson process with rate denoted by $\lambda(t \mid A(t), M(t), P(t), K; \gamma^\dagger) = 0.25(t + 0.05)\exp\{\gamma_8 A(t) + \gamma_9 M(t) + \gamma_{10} K_1 + \gamma_{11} K_2 + \gamma_{12} K_3 + \gamma_{13} P(t)\}$. We tested the four following combinations for the parameters $\gamma^\dagger$: 1)$\gamma^\dagger = (0, 0, 0, 0, 0, -5)$ (i.e., no bias due to the visit process expected); 2) $\gamma^\dagger = (0.5, 0.3, -0.5, -2, 0, -3)$; 3) $\gamma^\dagger = (0.5, -0.5, -0.2, -1, 1, -3)$; and 4) $\gamma^\dagger = (-1, -0.8, 0.1, 0.3, -1, -3)$. In a second set of simulation studies, we used Bernoulli random variables with probabilities denoted by $\lambda(t \mid A(t), M(t), P(t), K; \gamma)$ $= \text{expit}\{\gamma_1 + \gamma_2 A(t) + \gamma_3 M(t) + \gamma_4 K_1 + \gamma_5 K_2 + \gamma_6 K_3 + \gamma_7 P(t)\}$ to simulate the indicators. We tested the four following combinations for the parameters γ: 1)$\gamma = (0.4, 0, 0, 0, 0, 0, -5)$ (i.e., no bias due to the visit process expected); 2) $\gamma = (0.4, 1, -1, -0.5, -2, 0, -3)$; 3) $\gamma = (0.4, 0.5, -0.5, -0.2, -1, 1, -3)$; and 4) $\gamma = (0.4, -0.5, 0.8, 0.1, 0.3, -1, -3)$. In both sets of simulations, the observation indicators could be obtained by simulating Bernoulli random variables with probability proportional to the Poisson rate or the Bernoulli probability. When, at a given time point, the simulated observation indicator was simulated as 0, the corresponding outcome value was set to missing.

The ordinary least squares estimator, the inverse probability of treatment-weighted estimators and the FIPTM estimators were obtained by fitting standard linear regressions (e.g., with the function `lm` in R) using the weight statement whenever applicable for the inverse probability of treatment or the inverse intensity of visit weights. The conditional outcome mean models were obtained by fitting two different linear models, one conditioning on the confounders and the

treatment (corresponding to $\mu_a\{K, A = a; \hat{\alpha}_K\}$ in the main manuscript) and one conditional on the visit predictors and the treatment (corresponding to $\mu_a\{V, A = a; \hat{\alpha}_V\}$).

To assess the performance of the AAIIW estimator when the model $\mu_a\{K, A = a; \alpha_K\}$ is correctly specified, we had to know the true model $\mu_a\{K, A = a; \alpha_{K0}\}$. Based on the outcome generating mechanism, that true model is

$$\begin{aligned} E[Y(t) \mid A(t), K] &= E[\kappa + 1\ A(t) + 0.4\ K_1 + 0.05\ K_2 - 0.6\ K_3 \\ &\qquad + 3\{M(t) - E[M(t) \mid A(t), K_1, K_2, K_3]\} + 0.3\ P(t) + \epsilon(t) \mid A(t), K] \\ &= \kappa + A(t) + 0.4K_1 + 0.05K_2 - 0.6K_3 + 0.3P(t). \end{aligned}$$

The variable $P(t)$ is not mandatory to adjust for in our setting, since it should not affect the contrast in the outcome mean across treated ($A(t) = 1$) and untreated patients ($A(t) = 0$) (unless there was an interaction term between $P(t)$ and the treatment in the outcome generating mechanism). The correctly specified model $\mu_a\{V, A = a; \alpha_V\}$ merely contains all the predictors that were used in generating the outcome above (i.e., the treatment, the re-centered mediator, the pure predictor, and all the confounders).

Additional simulation with informative censoring

In additional simulations, we assessed the effect of informative censoring on the inference with the more naive estimators, the doubly-weighted estimator, and the proposed multiply robust estimator. We induced informative censoring by generating a censoring, binary indicator at each point in time, and removed any follow-up time (person-time) after the first indicator of censoring turned to 1.

We used an heterogeneous Bernoulli model to simulate the censoring indicators, with the probability of censoring given by $P(\xi_i(t) = 0 \mid \mathbf{V}_i(t); \boldsymbol{\phi}) = \exp(-5 + 0.8\ A_i(t) + 1\ M_i(t) - 0.6\ K_1 - 0.8\ K_2 - 0.5\ K_3)/\{1 + \exp(-5 + 0.8\ A_i(t) + 1\ M_i(t) - 0.6\ K_1 - 0.8\ K_2 - 0.5\ K_3)\}$. We tested this censoring mechanism coupled with two different vectors for the visit parameters (see Web Table 2 for details) and used, for simulating the visits, a Bernoulli model instead of Poisson since it was easier to parameterize.

In the results, we compared the performance of the same set of estimators as in the main simulation study, plus the same set of estimators that incorporated correctly specified IPCW (IPCW are discussed in the main text and in Web Appendix F - in summary, we cumulated the probability of still being observed as a function of patient characteristics up to any time point and used it in inverse weights, we also assessed a stabilizers which was modeled in the same way but as a function of baseline confounders only. The rest of the data generating mechanism was kept the same). The propensity score estimation and the outcome mean model conditional on the confounders were also adjusted for informative censoring by incorporating IPCW in their respective estimating equations, as discussed in Web Appendix F. Results of this additional analysis are discussed briefly in the main manuscript and a result table for the censoring simulation are shown in Web Appendix H.

Web Appendix H: Additional results from simulation studies

Web Figures 1, 2 and 3 and Web Tables 1 and 2 that contain simulation study results follow after the following discussion on the simulation results.

Lenghtier discussion on the main results from the simulation studies

The distributions of 1000 estimates obtained with each estimator using a sample of size 1000 patients are presented in Figure 1 in the main manuscript. The results are as expected. First, the OLS estimator is strongly biased in all γ parameter settings 1) to 4). In scenario 1) in which we expected no bias due to the visit process, the IPT-weighted estimator IPTc is empirically unbiased and the IPT-weighted estimator using a wrong treatment model, IPTnc, exhibits bias. In scenarios 2) to 4), both IPT-weighted estimators are biased since they do not account properly for the outcome observation process.

The doubly-weighted estimator DWc (which incorporates two correct weights) is consistently unbiased as it accounts properly for both types of bias. When the visit process is uninformative, in scenario 1) for the observation process, it is also unbiased even when the IIV weights are not correctly specified, as long as the IPT weights are correctly specified. In scenarios 2) to 4), the doubly-weighted estimator is only unbiased in settings in which its two weight models are correctly specified.

The multiply robust AAIIW estimator is empirically unbiased in all scenarios 1) to 4) for the observation process, whenever using one of the four combinations of correctly specified models shown in Table 1 or when all four nuisance models are correctly specified. It exhibits particularly small variance when the two conditional outcome mean models are correctly specified (scenario b from Table 1 in the main manuscript) or, as expected when all four models are correctly specified.

Simulation study results when using the Bernoulli model for the visits

Results for the second set of simulations using the Bernoulli probability to simulate the observations, and those for a sample of size 250 are presented in Web Figures 2 and 3. As expected, the estimators are more variable when using a sample size of 250, although the same patterns in the comparison of estimators are observed (Web Figures 1 and 3 correspond to sample sizes of 250 under a Poisson and a Bernoulli model for visits, respectively - similar results are observed with both).

The simulations using the Bernoulli probability did not require the use of Breslow's estimator for the baseline rate, which may partly explain the smaller variances observed overall (e.g., compare Figure 1 in the main manuscript and Web Figure 2).

Results for the simulation study with informative censoring

Results for the DGM with informative censoring that depended on the predictors of visit were as expected (Web Table 2). In the first visit scenario with $\gamma = (0.5, 0.3, -0.5, -2, 0.3, -3)$, informative censoring did not induce a lot of bias in the different estimators but the addition of a stabilizer to the weights helped in reducing the mean squared errors. In the second scenario with $\gamma = (0.5, -0.5, -0.2, -1, 1, -3)$ coupled with informative censoring, the informative censoring affected the empirical bias more (along with the mean squared errors). Adjustment via IPCW brought the estimates closer to the true causal effect, with a maximum bias that went from 0.31 to 0.14 after adjustment, for the AAIIW estimator. The AAIIW estimator coupled with IPCW performed particularly well when the two outcome conditional mean models were correctly specified, or when the outcome model conditional on the confounders and the IIV weights were correctly specified (bias smaller than 0.02 in all scenarios).

Additional results of the simulation studies: sample of size 250 with the use of a nonhomogeneous Poisson rate, or the use of a Bernoulli probability (sample sizes 250 and 1000) to simulate the observation indicators and Monte Carlo empirical bias and mean square error (MSE) in the simulation studies

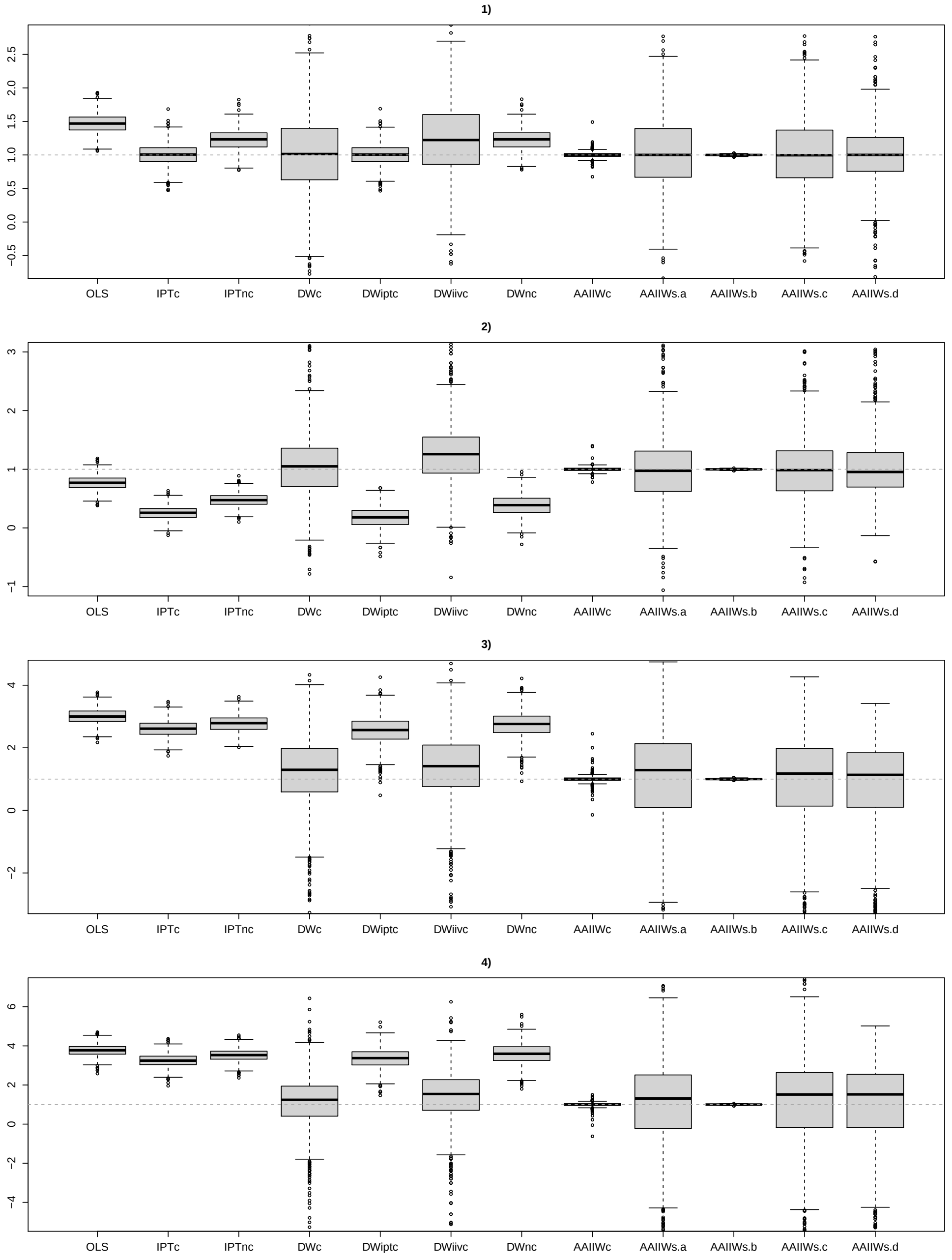

Web Figure 1: Results of the simulation studies with a sample size of 250 using a nonhomogeneous Poisson rate to simulate the observation indicators and the Andersen and Gill model with Breslow estimator to estimate the IIV weights. Each boxplot represents the distribution of 1000 estimates for the corresponding estimator. The dashed line represents the gold standard, i.e., the true value for the marginal effect of exposure that equals to 1. Different strengths of the visit process on covariates are represented with scenarios 1) $\gamma = (0, 0, 0, 0, 0, -5)$ (i.e., no bias due to the visit process expected); 2) $\gamma = (0.5, 0.3, -0.5, -2, 0, -3)$; 3) $\gamma = (0.5, -0.5, -0.2, -1, 1, -3)$; and 4) $\gamma = (-1, -0.8, 0.1, 0.3, -1, -3)$. OLS: Ordinary Least Squares; IPT: inverse probability of treatment weights; DW: Doubly-weighted estimator (corresponding to the FIPTM); AAIIW: The novel doubly augmented, doubly weighted estimator. The subscripts c, nc, $iptc$ and $iivc$ respectively mean all correct, all not correct, only IPT correct, and only IIV correct in the nuisance models. The subscripts $s.a$ to $s.d$ refer to scenarios a) to d) in Table 2 of the manuscript.

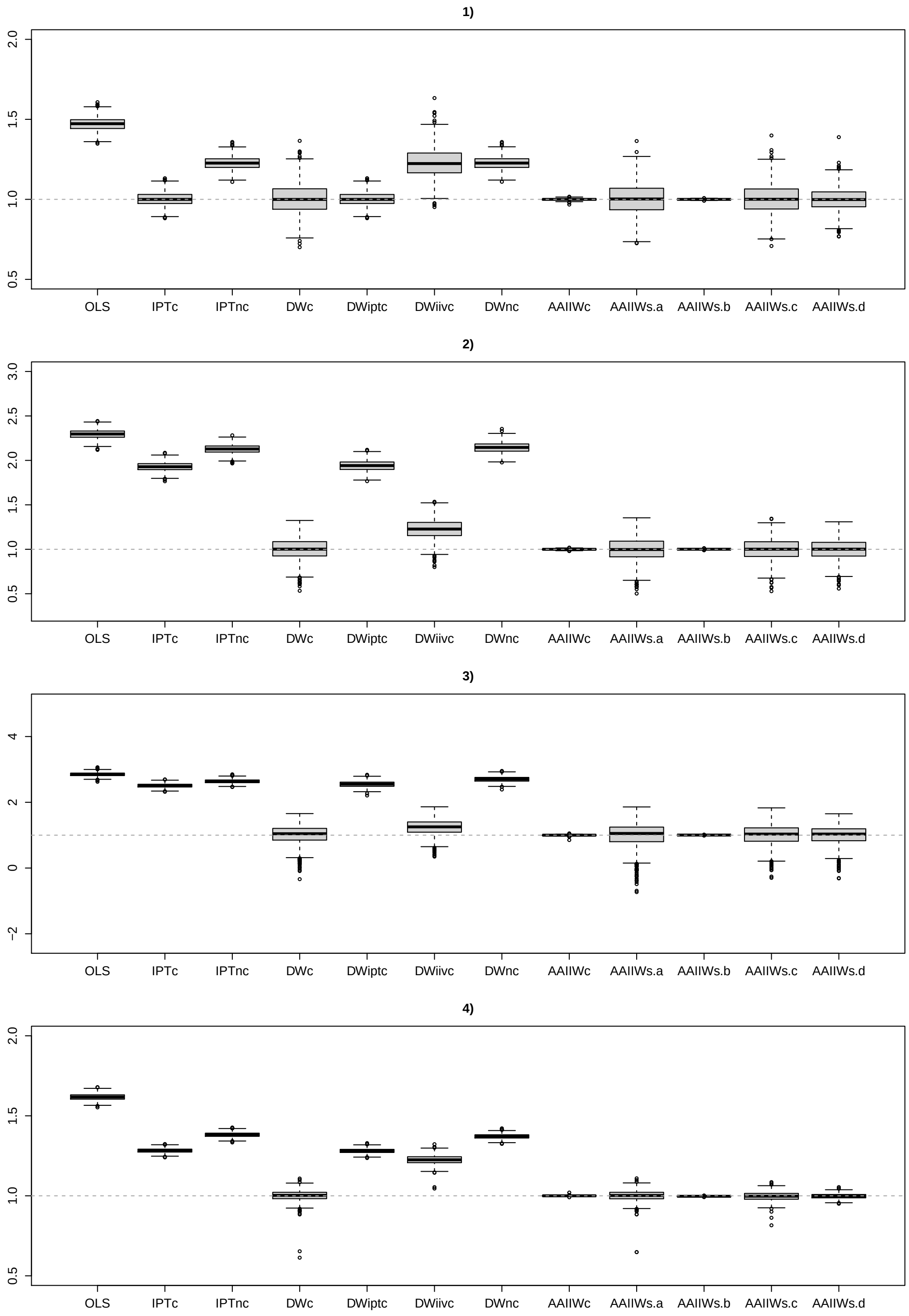

Web Figure 2: Results of the simulation studies using Binomial probabilities with a sample size of 1000. Each boxplot represents the distribution of 1000 estimates for the corresponding estimator. The dashed line represents the gold standard, i.e., the true value for the marginal effect of exposure that equals to 1. Different strengths of the visit process on covariates are represented with scenarios 1) $\gamma = (0.4, 0, 0, 0, 0, 0, -5)$ (i.e., no bias due to the visit process expected); 2) $\gamma = (0.4, 1, -1, -0.5, -2, 0, -3)$; 3) $\gamma = (0.4, 0.5, -0.5, -0.2, -1, 1, -3)$; and 4) $\gamma = (0.4, -0.5, 0.8, 0.1, 0.3, -1, -3)$. OLS: Ordinary Least Squares; IPT: inverse probability of treatment weights; DW: Doubly-weighted estimator (corresponding to the FIPTM); AAIIW: The novel doubly augmented, doubly weighted estimator. The subscripts *c*, *nc*, *iptc* and *iivc* respectively mean all correct, all not correct, only IPT correct, and only IIV correct in the nuisance models. The subscripts *s.a* to *s.d* refer to scenarios a) to d) in Table 2 of the manuscript.

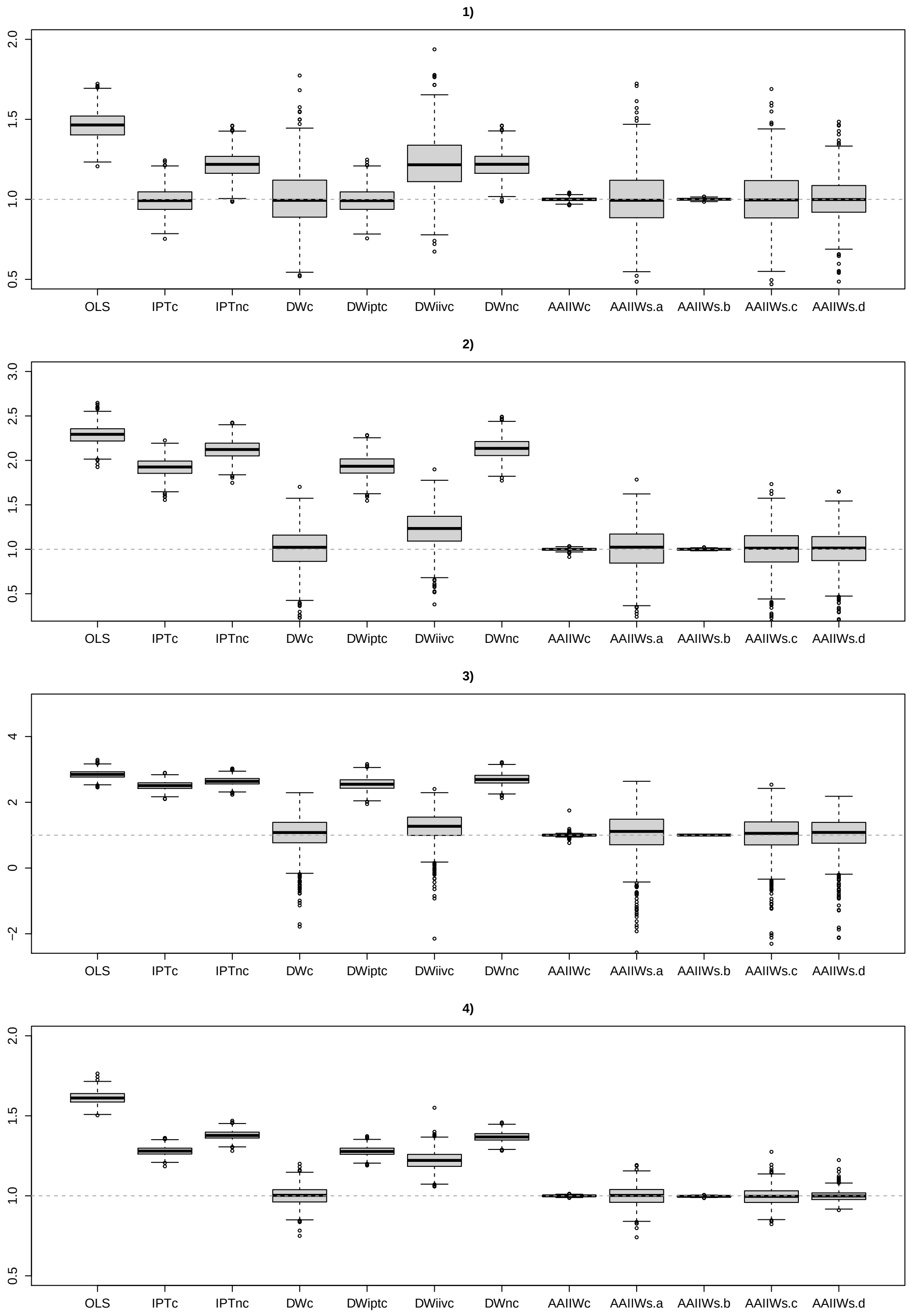

Web Figure 3: Results of the simulation studies using Binomial probabilities with a sample size of 250. Each boxplot represents the distribution of 1000 estimates for the corresponding estimator. The dashed line represents the gold standard, i.e., the true value for the marginal effect of exposure that equals to 1. Different strengths of the visit process on covariates are represented with scenarios 1) $\gamma = (0.4, 0, 0, 0, 0, 0, -5)$ (i.e., no bias due to the visit process expected); 2) $\gamma = (0.4, 1, -1, -0.5, -2, 0, -3)$; 3) $\gamma = (0.4, 0.5, -0.5, -0.2, -1, 1, -3)$; and 4) $\gamma = (0.4, -0.5, 0.8, 0.1, 0.3, -1, -3)$. OLS: Ordinary Least Squares; IPT: inverse probability of treatment weights; DW: Doubly-weighted estimator (corresponding to the FIPTM); AAIIW: The novel doubly augmented, doubly weighted estimator. The subscripts c, nc, $iptc$ and $iivc$ respectively mean all correct, all not correct, only IPT correct, and only IIV correct in the nuisance models. The subscripts $s.a$ to $s.d$ refer to scenarios a) to d) in Table 2 of the manuscript.

Web Table 1: Simulations results, empirical bias and mean squared error (MSE). OLS: Ordinary Least Squares; IPT: inverse probability of treatment weights; DW: Doubly-weighted estimator (corresponding to the FIPTM); AAIIW: The novel doubly augmented, doubly weighted estimator. The subscripts c, nc, $iptc$ and $iivc$ respectively mean all correct, all not correct, only IPT correct, and only IIV correct in the nuisance models. The subscripts $s.a$ to $s.d$ refer to scenarios a) to d) in Table 2 of the manuscript.

Estimator	Average $N(\tau)$ ($A_i(t)=0,1$) Poisson † or Bernoulli ‡	Gamma set γ Poisson † or Bernoulli ‡	Poisson rate				Bernoulli probability			
			$n=1000$		$n=250$		$n=1000$		$n=250$	
			Bias	MSE	Bias	MSE	Bias	MSE	Bias	MSE
OLS	(12, 12)†	1†	0.47	0.22	0.47	0.24	0.47	0.22	0.46	0.22
IPTc	(18, 18)‡	1‡	<0.01	0.01	0.01	0.03	<0.01	<0.01	0.01	0.01
IPTnc			0.23	0.06	0.23	0.08	0.23	0.05	0.22	0.05
DWc			0.01	0.13	0.01	0.44	<0.01	0.01	<0.01	0.03
DWiptc			<0.01	0.01	0.01	0.03	<0.01	<0.01	0.01	0.01
DWiivc			0.23	0.18	0.22	0.47	0.23	0.06	0.22	0.08
DWnc			0.23	0.06	0.23	0.08	0.23	0.05	0.22	0.05
AAIIWc			<0.01	<0.01	<0.01	<0.01	<0.01	<0.01	< 0.01	< 0.01
AAIIWs.a.			0.01	0.15	<0.01	0.73	<0.01	0.01	<0.01	0.03
AAIIWs.b.			<0.01	<0.01	<0.01	<0.01	<0.01	<0.01	<0.01	<0.01
AAIIWs.c.			<0.01	0.14	<0.01	0.84	<0.01	0.01	<0.01	0.03
AAIIWs.d.			<0.01	0.10	<0.01	0.46	<0.01	0.01	<0.01	0.02
OLS	(22, 17)†	2†	0.22	0.05	0.23	0.07	1.30	1.68	1.29	1.67
IPTc	(1, 7)‡	2‡	0.75	0.56	0.75	0.57	0.93	0.86	0.92	0.87
IPTnc			0.52	0.27	0.52	0.29	1.13	1.27	1.12	1.27
DWc			0.05	0.08	0.04	0.29	<0.01	0.02	<0.01	0.06
DWiptc			0.82	0.68	0.82	0.71	0.94	0.89	0.93	0.88
DWiivc			0.27	0.15	0.26	0.37	0.22	0.06	0.22	0.10
DWnc			0.61	0.38	0.61	0.41	1.14	1.31	1.13	1.30
AAIIWc			<0.01	<0.01	<0.01	<0.01	<0.01	<0.01	<0.01	<0.01
AAIIWs.a.			0.01	0.10	0.02	0.49	<0.01	0.02	<0.01	0.07
AAIIWs.b.			<0.01	<0.01	<0.01	<0.01	<0.01	<0.01	<0.01	<0.01
AAIIWs.c.			0.03	0.10	0.04	0.51	<0.01	0.02	<0.01	0.06
AAIIWs.d.			0.05	0.08	0.05	0.42	<0.01	0.01	<0.01	0.05
OLS	(3, 8)†	3†	2.02	4.09	2.00	4.08	1.85	3.43	1.85	3.43
IPTc	(6, 14)‡	3‡	1.62	2.63	1.61	2.65	1.51	2.27	1.51	2.28
IPTnc			1.79	3.22	1.78	3.23	1.64	2.69	1.64	2.70
DWc			0.01	0.67	0.18	1.63	0.01	0.09	0.04	0.29
DWiptc			1.56	2.59	1.55	2.62	1.55	2.41	1.55	2.44
DWiivc			0.21	0.57	0.34	1.55	0.23	0.12	0.24	0.28
DWnc			1.77	3.17	1.75	3.24	1.70	2.91	1.70	2.92
AAIIWc			<0.01	<0.01	<0.01	0.01	<0.01	<0.01	<0.01	<0.01
AAIIWs.a.			0.38	2.68	0.31	11.13	<0.01	0.14	<0.01	0.91
AAIIWs.b.			<0.01	<0.01	<0.01	<0.01	<0.01	<0.01	<0.01	<0.01
AAIIWs.c.			0.34	1.83	0.28	9.62	<0.01	0.10	0.01	0.42
AAIIWs.d.			0.45	1.80	0.43	8.67	<0.01	0.09	0.01	0.37
OLS	(2, 5)†	4†	2.78	7.77	2.78	7.80	0.62	0.38	0.61	0.38
IPTc	(81, 58)‡	4‡	2.26	5.12	2.25	5.19	0.28	0.08	0.28	0.08
IPTnc			2.54	6.45	2.53	6.48	0.38	0.15	0.38	0.14
DWc			0.01	0.84	0.10	1.93	<0.01	<0.01	<0.01	<0.01
DWiptc			2.35	5.57	2.35	5.79	0.28	0.08	0.28	0.08
DWiivc			0.25	0.91	0.37	2.08	0.23	0.05	0.22	0.05
DWnc			2.59	6.78	2.59	6.98	0.37	0.14	0.37	0.14
AAIIWc			<0.01	<0.01	<0.01	0.01	<0.01	<0.01	<0.01	<0.01
AAIIWs.a.			0.34	4.77	0.21	13.54	<0.01	<0.01	<0.01	<0.01
AAIIWs.c.			0.38	6.53	0.25	14.89	<0.01	<0.01	<0.01	<0.01
AAIIWs.d.			0.64	6.33	0.41	12.58	<0.01	<0.01	<0.01	<0.01

†. 1: γ=(0, 0, 0, 0, 0, -5); 2: γ=(0.5, 0.3, -0.5, -2, 0, -3); 3: γ=(0.5, -0.5, -0.2, -1, 1, -3); 4: γ=(-1, -0.8, 0.1, 0.3, -1, -3).
‡. 1: γ=(0.4, 0, 0, 0, 0, 0, -5); 2: γ=(0.4, 1, -1, -0.5, -2, 0, -3); 3: γ=(0.4, 0.5, -0.5, -0.2, -1, 1, -3); 4: γ=(0.4, -0.5, 0.8, 0.1, 0.3, -1, -3).

Web Table 2: Simulations results for the censoring study, sample size of 1000, 1000 simulations, without and with (w.) IPC stabilizer in the IPC weights, empirical bias and mean squared error (MSE). OLS: Ordinary Least Squares; IPT: inverse probability of treatment weights; DW: Doubly-weighted estimator (corresponding to the FIPTM); AAIIW: The novel doubly augmented, doubly weighted estimator. All the estimator names augmented with IPCW mean the inverse probability of censoring weighted estimators. The estimators without the IPCW acronym do not contain any informative censoring adjustment, i.e., their propensity score is not adjusted either for censoring. The subscript c means that the nuisance models used in these estimators are correctly specified. The subscripts $s.a$ to $s.d$ refer to scenarios a) to d) in Table 2 of the manuscript. The parameters for the censoring are given by (0.8, 1, -0.6, -0.8, -0.5, -3) for treatment, mediator, the three confounders, and the pure predictor, respectively. The dashed lines separate the estimators supposed to be non-convergent and convergent, only after IPC weighting.

		No IPC stabilizer		W. IPC Stabilizer	
	Visit mechanism	Bias	MSE	Bias	MSE
OLS	γ =(0.5, 0.3, -0.5, -2, 0.3, -3)	0.43	0.19	-	-
OLS-IPCW		0.05	0.34	0.05	0.02
IPTc		0.09	0.02	-	-
IPTc-IPCW		0.40	0.50	0.44	0.21
DWc		0.23	0.09	-	-
DWc-IPCW		0.01	0.42	0.01	0.04
AAIIWc		0.18	0.03	-	-
AAIIWc-IPCW		0.04	0.12	0.04	<0.01
AAIIWs.a.		0.20	0.09	-	-
AAIIWs.a.-IPCW		0.03	0.54	0.02	0.05
AAIIWs.b.		0.18	0.03	-	-
AAIIWs.b. -IPCW†		0.02	0.12	0.01	<0.01
AAIIWs.c.		0.18	0.08	-	-
AAIIWs.c.-IPCW†		0.01	0.55	0.01	0.05
AAIIWs.d.		0.19	0.04	-	-
AAIIWs.d.-IPCW		0.02	0.10	0.02	<0.01
OLS	γ =(0.5, -0.5, -0.2, -1, 1, -3)	1.97	3.91	-	-
OLS-IPCW		1.83	3.83	1.78	3.21
IPTc		1.62	2.64	-	-
IPTc-IPCW		1.51	2.73	1.46	2.15
DWc		0.28	0.37	-	-
DWc-IPCW		0.26	1.33	0.07	0.43
AAIIWc		0.29	0.09	-	-
AAIIWc-IPCW		0.18	0.14	0.13	0.02
AAIIWs.a.		0.31	0.73	-	-
AAIIWs.a.-IPCW		0.29	5.70	0.14	0.90
AAIIWs.b.		0.29	0.09	-	-
AAIIWs.b. -IPCW†		0.03	0.14	0.01	<0.01
AAIIWs.c.		0.30	0.73	-	-
AAIIWs.c.-IPCW†		0.16	6.15	0.02	0.97
AAIIWs.d.		0.29	0.09	-	-
AAIIWs.d.-IPCW		0.18	0.13	0.13	0.02

†. These estimators use wrongly specified propensity score. In this censoring study, we specified the wrong PS model as being the same as the right PS model but without the inverse probability of censoring weights (instead of wrong model meaning that it does not include all predictors, like in the main analysis).

Web Appendix I: Additional details on the Add Health Study

We applied the proposed AAIIW estimator and different more naive comparators to data from the *Add Health* study in the United States (Harris and Udry, 1994). It consists of a longitudinal study with multiple waves. The study started in 1994 when a pool of adolescents representative from the United States was selected. These adolescents became adults during the study. At each wave, they were asked to fill out in-home questionnaires (along with parents at the first wave). The data we used from the in-home questionnaires are publicly available. We did not consider the sampling weights in the following application.

We have access to data from the first four waves of the *Add Health* study, corresponding to the years 1994-1995, 1996, 2001-2002 and 2008-2009 respectively. For the purpose of this application, we consider that these four waves represent four consecutive time points, close in time, when observation could occur (even though these data do not consist of medical records, the missingness mechanism in these data is similar to that seen in medical records). Various types of information, such as demographics, health status, nutrition, family dynamics, sexual activity and substance use were collected for the study. Some questions varied across the four waves but we focused on a causal research question for which the exposure and outcome-related questions were asked at all four waves. Our goal was to estimate the marginal causal effect of counseling (psychotherapy) on alcohol consumption, based on one of the in-home questions, namely *In the past year, have you received psychological or emotional counseling?*. We think that the effect of counseling on alcohol consumption is mediated by the depressive mood of adolescents and that their mood can be affected by counseling and may in its turn affect alcohol consumption (see the assumed DGM in the main manuscript, Figure 1 (b)). Two important challenges we wished to consider in the analysis are the irregular observation of the outcome and, because the study is observational, the potential confounding of the psychotherapy-alcohol consumption relationship.

We selected potential confounders for that relationship, which included the teen's age, sex, socioeconomic status (SES), weight in pounds, and whether they smoked at least once in the previous month. The SES was computed by summing two variables that we transformed, namely the parents' total income in 1994 before taxes and one of the parents' education, usually that of the resident mother (Harris and Udry, 2002). The parent's total income was transformed into quintiles (1 to 5 with 5 being the highest). One of the parents' education was categorized in 5 levels corresponding to *1- 8th grade or less or never went to school*, *2-more than 8th grade but did not graduate high school*, *3-went to a business, trade or vocational school instead of high school, high school graduate, or completed a general educational development program*, *4–went to a business, trade or vocational school after high school, went to college but did not graduate*, or *5-graduated from college or university, or professional training beyond a 4-year college or university training.* The SES was defined as the sum of the two transformed 5-category variables.

The analysis dataset contained several missing values. Unless we had enough information in the dataset to replace missing values in the variables age and sex (e.g., if age was measured at a previous wave and it could be used for extrapolation), we used multiple imputations by chained equations (Rubin, 1988) five times, to impute missing values in these variables as well as in variables SES, smoking status, weight, depressive mood, and the exposure to counseling. We used these variables as predictors each time to impute each other variable using fully conditional specification. The alcohol outcome was kept as missing when it was not measured.

The outcome was defined using the question *Think of all the times you had a drink during the past 12 months. How many drinks did you usually have each time?*. It consists of a self-assessed number of drinks the adolescent would consume, on average, each time they consumed alcohol, ranging from 0 to 90. In this application, the outcome tended to be assessed at each of the four waves (i.e., not irregularly and with most data not being missing in the outcome). To assess the advantage of our approach, we simulated missingness in the outcome and assessed the different estimators in that setting, knowing the true underlying missingness mechanism. Assuming that all potential confounders as well as the mediator (depressive mood) and the exposure (counseling) affect the chance of observing the alcohol consumption outcome, the outcome observation (i.e., the opposite of missingness) was simulated using the following model across the four waves:

$$E[\mathrm{dN_i(t)} \mid \text{age, sex, counseling, depressive mood, SES}] = \text{expit}\{18-$$
$$0.3\ \text{age}(t) + 0.8\ \text{sex} + 1.8\ \text{counseling}(t) - 3\ \text{depressive mood}(t) - 1.3\ \text{SES}\}$$

for $t \in 1, 2, 3, 4$, where $\text{expit}(\cdot) = \exp(\cdot)/\left\{1 + \exp(\cdot)\right\}$.

We conducted the analysis using each of the five imputed datasets one by one. We used Rubin's rule (Rubin, 1976) to combine the final estimates from all the estimators compared, and 500 bootstrap samples to estimate their respective variance and obtain confidence intervals (CI). First, we fit a propensity score model as a function of age, sex, weight, SES and smoking. We fit two different proportional rate models for the observation of the outcome, one correctly specified (as a function of age, sex, counseling, depressive mood, and SES) and one that was not correctly specified (as a function of the sinus of age and the depressive mood, therefore not including the right format for the age variable and missing some important variables in the model). The estimators compared in the application are a standard ordinary least squares estimator (not adjusted for confounding nor for the observation process), an IIV-weighted estimator that does not account for confounding but does account for the observation process (we tested the two sets of the IIV weights), a doubly-weighted estimator corresponding to the FIPTM estimator (incorporating the IPT weights based on our assumptions on the potential confounders, and IIV weights - we tested the two sets of the IIV weights here again), and the AAIIW estimator in which we incorporated the IPT weights and the two different sets of the IIV weights, one at a time. We also added a complete data analysis in which an OLS, an IPT-weighted and an augmented IPW estimators were computed on the dataset with no missing data for the outcome. Results are presented in the main manuscript (Tables 3 and 4).

References

Harris, K. M., & Udry, J. R. (1994). National Longitudinal Study of Adolescent Health (Add Health), 1994-2002.

Rubin, D. B. (1976). Inference and missing data. Biometrika, 63(3), pp. 581-592.

Rubin, D. B. (1988). An overview of multiple imputation. In Proceedings of the survey research methods section of the American statistical association, 79, pp. 84. Princeton, NJ, USA: Citeseer.

Web Appendix J: Additional results in the Add Health Study

Tables of characteristics in the *Add Health* study stratified by weighting strategy

Web Table 3: Longitudinal characteristics stratified by adolescents receiving counselling therapy or not in the first dataset imputed with multiple imputations by chained equations, *Add Health* study, United States, 1996-2008

	Before inverse probability of treatment-weighting		After inverse probability of treatment-weighting	
Variable, N (%)	Counselling	No counselling	Counselling	No counselling
Age, mean (SD)	20.4 (5.8)	20.8 (5.7)	20.9 (6.0)	20.8 (5.7)
Female sex	1602 (60.7)	11,825 (50.6)	13,429 (51.6)	13,618 (52.3)
Weight, mean (SD)	155.1 (42.4)	160.8 (45.1)	160.4 (45.2)	160.2 (44.9)
Socioeconomic status, mean (SD)	6.6 (2.2)	6.6 (2.2)	6.6 (2.2)	6.6 (2.2)
Smoking	1200 (45.5)	6890 (29.5)	8024 (30.8)	8089 (31.1)
Depressive mood, mean (SD)†	1.9 (0.9)	1.4 (0.7)	1.8 (0.9)	1.4 (0.7)

†. This is not considered as a confounder but as a mediator in our analyses. Therefore, depressive mood was not included in the inverse probability of treatment weights. Acronym: SD, standard deviation.

Web Table 4: Longitudinal characteristics stratified by the alcohol consumption being observed or the full cohort follow-up whether the outcome is observed or not, in the first dataset imputed with multiple imputations by chained equations and after inducing outcome missingness, *Add Health* study, United States, 1996-2008

	Before inverse intensity of visit-weighting		After inverse intensity of visit-weighting	
Variable, N (%)	Observed	Entire follow-up	Observed	Entire follow-up
Age, mean (SD)	21.1 (5.7)	20.8 (5.7)	21.3 (5.7)	21.1 (5.7)
Female sex	12,455 (52.9)	13,427 (51.6)	12,044 (53.7)	12,455 (52.9)
Weight, mean (SD)	161.0 (45.2)	160.2 (44.9)	161.4 (45.4)	161.0 (45.3)
Socioeconomic status, mean (SD)	6.6 (2.2)	6.6 (2.2)	6.6 (2.2)	6.6 (2.2)
Smoking	7159 (30.4)	8090 (31.1)	6776 (30.2)	7180 (30.5)
Depressive mood, mean (SD)	1.3 (0.6)	1.4 (0.7)	1.3 (0.5)	1.3 (0.6)
Counselling	0.1 (0.3)	0.1 (0.3)	0.1 (0.3)	0.1 (0.3)

Acronym: SD, standard deviation.

Additional results in the analysis of the Add Health dataset:

In the first imputed dataset, we found an important difference in the proportion of males and females across both exposure groups, with females reporting higher rates of counseling, 16% more smokers in the counseling group than the other, and a greater depressive mood in those receiving counseling (Web Table 2). These variables were, therefore, considered potential confounders in our analysis, except for the mediator depressive mood that should not be conditioned upon in the confounding set. After IPT-weighting, the two exposure groups are similar with respect to all potential confounders (Web Table 2).

In the outcome observation model, using again the first imputed dataset as an example, we found modest differences in female sex and smoking status between those for whom the alcohol consumption was observed and the others (Web Table 3). After IIV weighting, most differences vanished, with female proportions and smoking status that were closer (Web Table 3).

In this application using data from the *Add Health* study, both the adjustment for confounding and the one for outcome missingness bring the estimates for the marginal effect of exposure to counseling towards the null (Tables 3 and 4 in the main manuscript). For instance, the IPT-weighted estimator is closer to 0 than the standard ordinary least squares estimator that does not adjust for confounding or the observation process. The estimator using the correctly adjusted IIV weights (IIV†) also brings the estimate towards the null when compared with using the wrong IIV weights (IIV‡). Combining both adjustments, the estimate for the causal marginal effect of counseling on alcohol consumption goes from 0.86 (95% CI 0.58, 1.10) with no adjustment at all,

to 0.40 (-1.36, 2.53) when using the correct IIV weights (and a propensity score based on our assumptions) in the multiply robust AAIIW estimator. Most importantly, the estimator that led to the closest estimates to the complete data analysis (point estimate 0.35 for the AIPW, Table 4) is the AAIIW estimator, which led to point estimates of 0.40 and 0.39 when using the correct or the wrong IIV weights, respectively. The FIPTM estimator, on the other hand, led to point estimates of 0.36 and 0.72, respectively (see Table 4), with the estimator using the wrong IIV weights being the one that led to the estimate further away from 0.35, considered to be the truth.

Those results indicate that in a setting in which we would not know the true observation mechanism, the AAIIW estimator might still lead to an estimate of the causal effect closer to the complete data analysis while we know that the FIPTM risks being biased when its weights are not correctly modelled. The AAIIW estimator might be protected against bias since its outcome mean model conditional on the observation predictors has a chance at being correctly specified.

Note, our proposed approach allows adjusting for previous (observed) treatments or outcomes as potential confounders or visit predictors, but it cannot address settings in which a previous outcome (that is not observed) affects the observation of any future outcome. In this application, since we have the complete data on the outcome before we induce missingness in the outcome process, we could have adjusted the IPT, IIV, or mean outcome models for all the previous outcome values if they were deemed to cause future visits, treatment prescription or outcomes. It was not tested, but could have removed additional residual confounding.

Web Appendix K: Recommendations for choosing adjustment sets

Additional recommendations for the choice of confounders and visits predictors sets:

An analyst using the novel AAIIW approach, in collaboration with an expert from the substantive research field, should identify the confounders of the relationship between the exposure and outcome and the outcome observation predictors at risk of creating spurious associations between the exposure and the outcome (conditional exchangeability assumption). The use of a causal diagram can help in depicting the open paths by which dependencies that are not due to causal effects arise. It is not enough to include these variables in the weight models (IPT or IIV weights) or in the conditional outcome mean models discussed in this manuscript. A model that is correctly specified implies that the functional format of the predictors in the model is correctly specified (see Web Appendix D for more details on correct specification).

Second, the analyst should ensure that the observations included in the analysis meet the two positivity assumptions for the treatment and the observation models. They might decide to remove from the analysis a patient with a set of characteristics that are only represented in one of the two exposure groups (otherwise, the positivity of treatment assumption may be violated) or with a set of characteristics that are only represented when the outcome is not observed (otherwise violating the positivity of observation assumption). Third, the causal assumption of consistency of the outcome must be met. In our application to the *Add Health* study, we assume that the outcome observed in those who claimed to have received counseling is truly equal to their potential outcome under counseling, and vice-versa. Measurement errors, for instance, patients not having filled in the information correctly, could alter the estimates.
