# Supplementary material for: Multiply robust estimation of marginal structural models in observational studies subject to covariate-driven observations
Source: Biometrics. 2024 Jul 16;80(3):ujae065. doi: 10.1093/biomtc/ujae065 (PMC11250490; doi:10.1093/biomtc/ujae065)
Supplement: ujae065_Supplemental_Files — Web Appendices A, B, C, D, E, and F referenced in Section 2, Web Appendices G and H referenced in Section 3, Web Appendices I and J referenced in Section 4, Web Appendix K referenced in Section 5, and the R code to reproduce the simulation studies from Section 3 are available with this paper at the Biometrics website on Oxford Academic. [file ujae065_supplemental_files.zip › R Code Coulombe and Yang 2023.pdf]

```
##############################
## For manuscript entitled
## 'Multiply robust estimation of marginal structural models in observational
studies subject to covariate-driven
    observations '
## Authors Janie Coulombe and Shu Yang
## April 2023
##############################



rm(list=ls(all=TRUE))

## Packages to download
library(survival)
library(rootSolve)

# Sample size
sampsize<-  1000 # 250

# Nb simulations
nbsimul<- 1000

# Choose the set of gamma parameters for the observation of the outcome process
gammaset<- 1 # 2, 3, or 4

# Max follow-up time
TAU<-2

## Treatment model parameters
    ..beta0=-0.5; ..beta1=+0.8; ..beta2=-0.4; ..beta3= -0.4;

## For defining the 3 confounders "age, male, height"
    ..agemean=1;  ..agesd=1;  ..p_male=0.55; ..heightmean=0; ..heightsd=1;

## Parameters related to mediator Z
    ..mu1d=4; ..sigma2_1d=2; ..mu2d=2; ..sigma2_2d=1; ..betaZ<- 3

## Parameters outcome model
    # age           sex            height
    ..beta4<-0.4; ..beta5<-0.05; ..beta6<- -0.6;
    # Q            treatment
    ..bq<- 0.3;    ..betaX<- 1

## For error term in outcome model
    ..sigmaepsilond<- 0.1; ..meanphid=0; ..sigmaphid=0.2;

## To save the proportion of rates censored at 1
## (the visits are simulated using a Bernoulli with prob proportional to rate so
it must be between
  ## 0, 1) -- keep track of proportion
   trunc<-c()

## Gamma set parameters

     if (gammaset==1){
        ..gamma1<- 0;     ..gamma2<- 0; ..gamma3<-  0; ..gamma4<- 0
        ..gamma5<- 0;      ..gamma6<- -5 }

    if (gammaset==2){
        ..gamma1<-  0.5;  ..gamma2<- 0.3; ..gamma3<- -0.5; ..gamma4<- -2
        ..gamma5<- 0;    ..gamma6<- -3  }
```

```
    if (gammaset==3){
        ..gamma1<- 0.5;  ..gamma2<- -0.5; ..gamma3<- -0.2 ; ..gamma4<- -1
        ..gamma5<- 1 ;    ..gamma6<- -3   }

    if (gammaset==4){
        ..gamma1<- -1;  ..gamma2<- -0.8; ..gamma3<- 0.1 ; ..gamma4<- 0.3
        ..gamma5<- -1 ;    ..gamma6<- -3   }


    # data.frames to store the results, return at end of function
    coefMat <- data.frame(matrix(nr=nbsimul, nc=1*12))
    names(coefMat) <- c("OLS"
                       ,"IPT_c"
                       ,"IPT_nc"
                       ,"DW_iptc_iivc"
                       ,"DW_iptc_iivnc"
                       ,"DW_iptnc_iivc"
                       ,"DW_iptnc_iivnc"
                       ,"MAR_cccc"
                       ,"uniMAR_twoweights_1"
                       ,"uniMAR_twocond_1"
                       ,"uniMAR_iivcondkOK_1"
                       ,"uniMAR_iptcondvOK_1"      )

    BIASmat <- data.frame(matrix(nr=nbsimul, nc=12))

    names(BIASmat) <- c("OLS"
                       ,"IPT_c"
                       ,"IPT_nc"
                       ,"DW_iptc_iivc"
                       ,"DW_iptc_iivnc"
                       ,"DW_iptnc_iivc"
                       ,"DW_iptnc_iivnc"
                       ,"MAR_cccc"
                       ,"uniMAR_twoweights_1"
                       ,"uniMAR_twocond_1"
                       ,"uniMAR_iivcondkOK_1"
                       ,"uniMAR_iptcondvOK_1"      )

   # to gather statistics on visits
    visitMat <- data.frame(matrix(nr=nbsimul, nc=3))
    names(visitMat) <- c("mVisit","mXtot","mXVisit")


   for(S in 1  : nbsimul){

      # print the simulation number
      print(S)


        N.pers <- sampsize
        N.times <- TAU*100
        N.tot <- N.pers*N.times
        mat<- data.frame(matrix(NA, nrow=N.tot, ncol= 13))

        names(mat)<-
        c('ID','time','X','Y','age','sex','height','Q','visit','Z', 'Zm',
'ratei', 'pt')
        #  1      2    3   4    5     6       7     8     9    10    11      12
13

        mat[,'ID']     <- rep(1:N.pers, each=N.times)
        mat[,'time']   <- rep(1:N.times/100,   N.pers)
```

```
       ## Confounders generation

        mat[,'age']    <-
            rep(rnorm(mean=..agemean,    sd=..agesd,    n=N.pers),each=N.times)
        mat[,'sex']    <-
            rep(rbinom(p=..p_male,       size=1,        n=N.pers),each=N.times)
        mat[,'height'] <-
            rep(rnorm(mean=..heightmean, sd=..heightsd, n=N.pers),each=N.times)



        ## Treatment generation

        p_treated <-
           exp(..beta0+ ..beta1*mat$age+ ..beta2*mat$sex+ ..beta3*mat$height)/
        (1+exp(..beta0+ ..beta1*mat$age+ ..beta2*mat$sex+ ..beta3*mat$height))

        mat[,'X']      <- rbinom(p=p_treated, size=1, n=N.tot)



        ## Mediator generation

        Z1a <- rnorm(mean=..mu1d, sd=sqrt(..sigma2_1d), n=N.tot) ## N(4,2)
        Z1b <- rnorm(mean=..mu2d, sd=sqrt(..sigma2_2d), n=N.tot) ## N(2,1)
        mat[,'Z']      <- ifelse(mat$X==1,Z1b,Z1a)
        mat[,'Zm']     <- rep(NA, N.tot)
        mat[,'Q']   <- rnorm(mean=0.5, sd=0.3, n=N.tot)

        alpha0  <- 0.5
        phi     <-rnorm(mean=..meanphid, sd=..sigmaphid,     n=N.tot)
        epsilon <-rnorm(mean=phi       , sd=..sigmaepsilond, n=N.tot)
        center  <-ifelse(mat$X==1, ..mu2d, ..mu1d)  # 2, 4
        centerZ<- predict( lm(mat[,'Z'] ~ mat[,'age'] + mat[,'sex'] +
mat[,'height'] + mat[,'X'] ), type='response')
             ## Expectation of Z given age, sex, height


        ## Outcome generation
        mat[,'Y'] <- alpha0 +
               ..betaX*mat$X +
               ..beta4*mat$age + ..beta5*mat$sex + ..beta6*mat$height +
               ..betaZ*(mat$Z - centerZ) +
               ..bq*mat$Q +
               epsilon

        expit<- function(yy){exp(yy)/(1+exp(yy))}

       ## Observation rate
       ratei<- 0.25*(mat[,'time']+0.05) *  exp(  ..gamma1*mat[,'X']
+ ..gamma2*mat[,'Z']   +
                     ..gamma3*mat[,'age'] + ..gamma4*mat[,'sex']
+ ..gamma5*mat[,'height'] +
                      ..gamma6*mat[,'Q'] )

        mat[,'ratei']<- ratei
        mat[,'pt']<- p_treated
        trunc<- append( trunc, sum(ratei[ratei>1])/length(ratei)*100)
        ratei[ratei>1]<- 0.9999999999999 # remove rates higher than 1 as a
probability of jump
```

```
        ## Observation indicator
        mat[,'visit']<-  rbinom(prob= ratei, size=1, n=N.tot)


        # data generation finished
        # ========================================================================



        ##############################
        ## Compute Estimator         #
        ##############################




        ## Keep only the visits

        Data <- mat
        Data[Data$visit==0,]$Y<-NA ## Put outcome as missing if no visit


        ## Visit intensity model (good model)

        Data$t1<-Data$time-0.01
        Data$t2<-Data$time
        coxfit<- coxph( Surv(t1,t2, visit) ~ X+Z+age+sex+height+Q , data=Data)
        gamma  <- coxfit$coef

        ## One other model, for the wrong visit model:
        gamma2b<-coxph(Surv(t1,t2,visit)~ sex+height , data=Data)$coef

        ## compute the baseline hazard
        baseh<- basehaz(coxfit, centered=FALSE)
        colnames(baseh)[2]<- 'time2'
        haz <-   baseh[,1][-1] - baseh[,1][- (length(baseh[,1]))]
        baseh$col3<- round(baseh[,2],2)
        baseh$haz<- c(baseh$hazard[1],haz)
        Data$col3<- round(Data$time,2)
        dat1<- merge( baseh, Data,   all.y=TRUE, by= 'col3')
        Data <- dat1[order(   dat1$ID, dat1$time  ),]


        ## Right IIV weights
        Data$rho_i<- Data$haz* exp(   gamma[1]*Data$X +   gamma[2]*Data$Z +
                                gamma[3]*Data$age + gamma[4]*Data$sex  +
gamma[5]*Data$height +
                                    gamma[6]*Data$Q)
        ## Wrong IIV weights
        Data$rho_i2<-   exp(gamma2b[1]*Data$sex + gamma2b[2]*Data$height)

        # Compute the propensity scores and two types of IPTW (one correct
        # iptw, one wrong iptw2):

        Data$Ages<- (Data$age)^2
        ## Correct
        Data$ps <-predict(glm(X ~ age + sex + height, data=Data,
family='binomial'),type='response')
        a1sups<- 1/Data$ps
        a1supms<- 1/(1-Data$ps)
        Data$iptw <-  ifelse(Data$X==1, a1sups ,a1supms )
        ## Wrong
        Data$ps2<-predict(glm(X ~ age , data=Data,
family='binomial'),type='response')
        Data$iptw2<- 1/ifelse(Data$X==1, Data$ps2, (1-Data$ps2))
```

```
	#########################
	## standard estimators ##
	#########################


	## OLS ESTIMATOR ## observation prosess ignored
	OLS <-lm(Y~ 1+ X, data=Data)$coef[2]

	## IPT ESTIMATOR, weight OK ## observation prosess ignored,
	IPT_c <-lm(Y~ 1+ X , weight=iptw  , data=Data)$coef[2]

	## IPT ESTIMATOR, weight not OK ## observation prosess ignored,
	IPT_nc <-lm(Y~ 1+ X , weight=iptw2  , data=Data)$coef[2]

	## DOUBLY WEIGHTED all correct weights ##
	DW_iptc_iivc <- lm(Y~ 1 + X , weight=iptw *1/rho_i , data=Data)$coef[2]

	## DOUBLY WEIGHTED iiv not correct ##
	DW_iptc_iivnc <- lm(Y~ 1 + X , weight=iptw *1/rho_i2 , data=Data)
$coef[2]

	## DOUBLY WEIGHTED ipt not correct ##
	DW_iptnc_iivc <- lm(Y~ 1 + X , weight=iptw2 *1/rho_i , data=Data)
$coef[2]

	## DOUBLY WEIGHTED both weights are wrong ##
	DW_iptnc_iivnc <- lm(Y~ 1 + X , weight=iptw2 *1/rho_i2 , data=Data)
$coef[2]


	## Correct conditional outcome mean models for AAIIW
	Data$Zmc<- Data$Z  -  predict( lm( Z ~ X + age + sex + height,
data=Data), type='response')

	## Conditional on visit predictors
	allcoefs<-   lm( Y ~  1 + Q + Zmc +  X + age + sex + height ,  data=Data
)
	Data1<- Data
	Data0<- Data
	Data1$X<- 1
	Data0$X<- 0

	Data$predv0<- predict(allcoefs, Data0)
	Data$predv1<- predict(allcoefs, Data1)

	## conditional on confounders
	allcoefs2<-   lm( Y ~  X + age + sex + height + Zmc  ,  data=Data )
	Data1<- Data
	Data0<- Data
	Data1$X<- 1
	Data0$X<- 0
	Data$predk0<- predict(allcoefs2, Data0)
	Data$predk1<- predict(allcoefs2, Data1)


	## Wrong conditional outcome models

	# Model E(Y given V, treatment a) WRONG
	allcoefs3<-   lm( Y ~  1 +   X + age  + height ,  data=Data )
	Data$predv02<- predict(allcoefs3, Data0)
	Data$predv12<- predict(allcoefs3, Data1)
```

```
       # Model E(Y given K, treatment a) WRONG
       allcoefs4<-   lm( Y ~  1 + X+ height ,  data=Data )
       Data$predk02<- predict(allcoefs4, Data0)
       Data$predk12<- predict(allcoefs4, Data1)

       # For the root solver to work:
       data<- Data
       ## Replace missing Y by 0 so that the root solving works (will not change
the estimate
        ## since it only sums the Y when dN=1)
       data[is.na(data$Y)==1,]$Y <- 0
       ## Remove times when there was never any visit otherwise the root solving
function
        ## does not work, because of divisions by 0
       data<-  data[data$rho_i!=0,]

       ###########################################
       ### AAIIW estimator (all versions follow) #
       ###########################################

       ####################
       # All good models  #
       ####################

       pst<- data$ps
       predk0g<- data$predk0
       predk1g<- data$predk1
       ro<- data$rho_i
       predv0g<-data$predv0
       predv1g<-data$predv1

       ## Estimate beta0 first
       MAR_cccc_beta0pp <-  function(beta0){
            mean(        (       (1-data$X)/(1-pst)*data$Y -
                         (  (1-data$X) - (1-pst) ) /(1-pst)*predk0g -
beta0 )*data$visit/ro  ) -
                    mean(     (   (1-data$X)/(1-pst)*predv0g -
                    (  (1-data$X) - (1-pst) ) /(1-pst)*predk0g -
beta0  )*(data$visit-ro)/ro)
                                    }

       beta0p <- uniroot(MAR_cccc_beta0pp, c(-100,100))$root

       ##Plug beta0p in the other estimating equation here to get beta1:

        MAR_cccc_beta1p<- function(beta1){
            mean(        (       (data$X)/(pst)*data$Y -
                         (  (data$X) - (pst) ) /(pst)*predk1g - beta0p -
beta1 )*data$visit/ro  ) -
                    mean(     (   (data$X)/(pst)*predv1g -
                     (  (data$X) - (pst) ) /(pst)*predk1g - beta0p -
beta1 )*(data$visit-ro)/ro)
                                    }
        uniMAR_cccc_1 <- uniroot(MAR_cccc_beta1p, c(-100,100))$root

       ##################################################
       ### AAIIW with two correctly specified weights ###
       ##################################################

       pst<- data$ps
       ro<- data$rho_i

       predk0g<- data$predk02
```

```
       predk1g<- data$predk12

       predv0g<-data$predv02
       predv1g<-data$predv12

       ## Beta0 first
       MAR_cccc_beta0q <-  function(beta0){
            mean(        (       (1-data$X)/(1-pst)*data$Y -
                         (  (1-data$X) - (1-pst) ) /(1-pst)*predk0g -
beta0 )*data$visit/ro  ) -
                  mean(     (   (1-data$X)/(1-pst)*predv0g -
                  (  (1-data$X) - (1-pst) ) /(1-pst)*predk0g -
beta0  )*(data$visit-ro)/ro)
                                 }
       beta0q <- uniroot(MAR_cccc_beta0q, c(-100,100))$root

       ## beta1 estimation (causal effect)
       MAR_cccc_beta1q<- function(beta1){
            mean(        (       (data$X)/(pst)*data$Y -
                         (  (data$X) - (pst) ) /(pst)*predk1g - beta0q -
beta1 )*data$visit/ro   ) -
                  mean(     (   (data$X)/(pst)*predv1g -
                  (  (data$X) - (pst) ) /(pst)*predk1g - beta0q -
beta1 )*(data$visit-ro)/ro)
                                    }
        uniMAR_twoweights_1 <- uniroot(MAR_cccc_beta1q, c(-100,100))$root


      ##############################################################
      ### AAIIW with two correctly specified cond. outcome models ##
      ##############################################################

       pst<- data$ps2
       ro<- data$rho_i2

       predk0g<- data$predk0
       predk1g<- data$predk1

       predv0g<-data$predv0
       predv1g<-data$predv1

       ## beta0
       MAR_cccc_beta0t <-  function(beta0){
            mean(        (       (1-data$X)/(1-pst)*data$Y -
                         (  (1-data$X) - (1-pst) ) /(1-pst)*predk0g -
beta0 )*data$visit/ro   ) -
                  mean(     (   (1-data$X)/(1-pst)*predv0g -
                  (  (1-data$X) - (1-pst) ) /(1-pst)*predk0g -
beta0  )*(data$visit-ro)/ro)
                                    }

        beta0t <- uniroot(MAR_cccc_beta0t, c(-100,100))$root

       ## beta 1 estimation
        MAR_cccc_beta1t<- function(beta1){
            mean(        (       (data$X)/(pst)*data$Y -
                         (  (data$X) - (pst) ) /(pst)*predk1g - beta0t -
beta1 )*data$visit/ro  ) -
                  mean(     (   (data$X)/(pst)*predv1g -
                  (  (data$X) - (pst) ) /(pst)*predk1g - beta0t -
beta1 )*(data$visit-ro)/ro)
                                    }
        uniMAR_twocond_1 <- uniroot(MAR_cccc_beta1t, c(-100,100))$root
```

```
######################################################################
### AAIIW with correct IIV and correct cond. outcome on confounders ##
######################################################################

pst<- data$ps2
ro<- data$rho_i

predk0g<- data$predk0
predk1g<- data$predk1

predv0g<-data$predv02
predv1g<-data$predv12

## beta0
MAR_cccc_beta0l <-  function(beta0){
      mean(        (       (1-data$X)/(1-pst)*data$Y -
                   (  (1-data$X) - (1-pst) ) /(1-pst)*predk0g -
beta0 )*data$visit/ro  ) -
             mean(     (   (1-data$X)/(1-pst)*predv0g -
             (  (1-data$X) - (1-pst) ) /(1-pst)*predk0g -
beta0  )*(data$visit-ro)/ro)
                                }

beta0l <- uniroot(MAR_cccc_beta0l, c(-100,100))$root

## beta1 estimation
MAR_cccc_beta1l<- function(beta1){
      mean(        (       (data$X)/(pst)*data$Y -
                   (  (data$X) - (pst) ) /(pst)*predk1g - beta0l -
beta1 )*data$visit/ro   ) -
             mean(     (   (data$X)/(pst)*predv1g -
             (  (data$X) - (pst) ) /(pst)*predk1g - beta0l -
beta1 )*(data$visit-ro)/ro)
                                }
uniMAR_iivcondkOK_1 <- uniroot(MAR_cccc_beta1l, c(-100,100))$root



######################################################################
      ### AAIIW with correct IPT and correct cond. outcome on visit predictors
##

######################################################################

 pst<- data$ps
 ro<- data$rho_i2

 predk0g<- data$predk02
 predk1g<- data$predk12

 predv0g<-data$predv0
 predv1g<-data$predv1

 ## beta0
 MAR_cccc_beta0f <-  function(beta0){
     mean(        (       (1-data$X)/(1-pst)*data$Y -
                   (  (1-data$X) - (1-pst) ) /(1-pst)*predk0g -
beta0 )*data$visit/ro   ) -
             mean(     (   (1-data$X)/(1-pst)*predv0g -
             (  (1-data$X) - (1-pst) ) /(1-pst)*predk0g -
beta0  )*(data$visit-ro)/ro)
                                                    }
```

```
       beta0f <- uniroot(MAR_cccc_beta0f, c(-100,100))$root

       ## beta1 estimation
        MAR_cccc_beta1f<- function(beta1){
             mean(        (       (data$X)/(pst)*data$Y -
                         (  (data$X) - (pst) ) /(pst)*predk1g - beta0l -
beta1 )*data$visit/ro  ) -
                   mean(     (   (data$X)/(pst)*predv1g -
                   (  (data$X) - (pst) ) /(pst)*predk1g - beta0l -
beta1 )*(data$visit-ro)/ro)
                                 }

        uniMAR_iptcondvOK_1 <- uniroot(MAR_cccc_beta1f, c(-100,100))$root




   ###### Record the values of each estimate, for each estimator

        coefMat[S,] <- c(OLS
                       ,IPT_c
                       ,IPT_nc
                       ,DW_iptc_iivc
                       ,DW_iptc_iivnc
                       ,DW_iptnc_iivc
                       ,DW_iptnc_iivnc
                       ,uniMAR_cccc_1
                       ,uniMAR_twoweights_1
                       ,uniMAR_twocond_1
                       ,uniMAR_iivcondkOK_1
                       ,uniMAR_iptcondvOK_1)
   }



 # Obtain the summary for of each estimator
summary(coefMat)
```
