# Supplementary material for: Multiply robust estimation of marginal structural models in observational studies subject to covariate-driven observations
Source: Biometrics. 2024 Jul 16;80(3):ujae065. doi: 10.1093/biomtc/ujae065 (PMC11250490; doi:10.1093/biomtc/ujae065)
Supplement: ujae065_Supplemental_Files — Web Appendices A, B, C, D, E, and F referenced in Section 2, Web Appendices G and H referenced in Section 3, Web Appendices I and J referenced in Section 4, Web Appendix K referenced in Section 5, and the R code to reproduce the simulation studies from Section 3 are available with this paper at the Biometrics website on Oxford Academic. [file ujae065_supplemental_files.zip › README.pdf]

The R code in the file <R Code Coulombe and Yang 2023.txt> can be used to reproduce the simulation studies from the manuscript entitled

''Multiply robust estimation of marginal structural models in observational studies subject to covariate-driven observations''

from authors Janie Coulombe (Universite de Montreal) and Shu Yang (North Carolina State University).

You can contact author JC for more details or questions about the code (email: janie.coulombe@umontreal.ca).

--

The structure of the R code goes (broadly) as follows:

- Definition of the parameters
- Simulation of the longitudinal dataset for the entire cohort
- Remove the outcome values at times when there is no visit
- Compute estimators:
    - compute right and wrong IIV weights
    - compute right and wrong propensity scores for IPT weights
    - compute OLS, IPT, and doubly-weighted (DW) estimators with different combinations of right or wrong inverse weights
    - also fit the outcome conditional mean models
    - compute the AAIIW estimator under different combinations of right or wrong inverse weights and outcome mean models

  All estimators are recorded in a row of coefMat[S,] where S represents the simulation number
